# TANK-binding kinase 1 (TBK1) modulates inflammatory hyperalgesia by regulating MAP kinases and NF-κB dependent genes

**DOI:** 10.1186/s12974-015-0319-3

**Published:** 2015-05-23

**Authors:** Christine V. Möser, Heike Stephan, Katharina Altenrath, Katharina L. Kynast, Otto Q. Russe, Katrin Olbrich, Gerd Geisslinger, Ellen Niederberger

**Affiliations:** Pharmazentrum Frankfurt/ZAFES, Institut für Klinische Pharmakologie, Klinikum der Goethe-Universität Frankfurt, Theodor Stern Kai 7, 60590 Frankfurt am Main, Germany

**Keywords:** I-κB Kinases, TBK1, Nociception, Inflammation, c-fos

## Abstract

**Background:**

TANK-binding kinase (TBK1) is a non-canonical IκB kinase (IKK) involved in the regulation of type I interferons and of NF-κB signal transduction. It is activated by viral infections and inflammatory mediators and has therefore been associated with viral diseases, obesity, and rheumatoid arthritis. Its role in pain has not been investigated so far. Due to the important roles of NF-κB, classical IκB Kinases and the IKK-related kinase, IKKε, in inflammatory nociception, we hypothesized that TBK1, which is suggested to form a complex with IKKε under certain conditions, might also alter the inflammatory nociceptive response.

**Methods:**

We investigated TBK1 expression and regulation in “pain-relevant” tissues of C57BL/6 mice by immunofluorescence, quantitative PCR, and Western blot analysis. Furthermore, nociceptive responses and the underlying signal transduction pathways were assessed using TBK1^−/−^ mice in two models of inflammatory nociception.

**Results:**

Our data show that TBK1 is expressed and regulated in the spinal cord after peripheral nociceptive stimulation and that a deletion of TBK1 alleviated the inflammatory hyperalgesia in mice while motor function and acute nociception were not altered. TBK1-mediated effects are at least partially mediated by regulation of NF-κB dependent COX-2 induction but also by alteration of expression of c-fos via modulation of MAP kinases as shown in the spinal cord of mice and in cell culture experiments.

**Conclusion:**

We suggest that TBK1 exerts pronociceptive effects in inflammatory nociception which are due to both modulation of NF-κB dependent genes and regulation of MAPKs and c-fos. Inhibition of TBK1 might therefore constitute a novel effective tool for analgesic therapy.

**Electronic supplementary material:**

The online version of this article (doi:10.1186/s12974-015-0319-3) contains supplementary material, which is available to authorized users.

## Background

TANK-binding kinase 1 (TBK1, NAK1, T2K) constitutes an IκB kinase (IKK)-related kinase which has been associated with interferon regulatory factor (IRF)- and nuclear factor (NF)-κB-activation [1]. In this context, it is involved in innate immune defense and its dysregulation has been described in the context of several diseases, particularly inflammatory disorders and cancer. So far, it is not clear which of the TBK1-driven pathways is functionally active in different diseases. Several publications suggest that TBK1 is able to act in context with its potential binding partner IκB kinase epsilon (IKKε) [2–4]. On the other hand, it is proposed to be an independent kinase [5, 6], a suggestion that is favored by constitutive TBK1 expression in many cells while IKKε must be induced by a variety of stimuli in most cells. To date, it appears that pain, inflammation, and cancer are associated with IKKε- or TBK1-specific NF-κB pathway activation while IRFs and the IFN pathways are involved in pathologies such as rheumatic diseases [7]. The classical NF-κB activation cascade has frequently been associated with the development of pathophysiological pain (reviewed in [8]). Depletion of NF-κB p50 as well as inhibition or knock-out of classical IKKs resulted in reduced nociceptive responses due to a decreased expression of NF-κB-regulated genes [9–12]. We recently showed that IKKε affects inflammatory nociception also by modulation of NF-κB rather than IRF activity. Deletion of IKKε in mice reduced the nociceptive behavior in inflammatory models associated with a loss of inflammation-evoked NF-κB activation and a decreased induction of NF-κB dependent proinflammatory genes while IRF-3 was not altered. Knock-down of IKKε by siRNA indicated that impaired phosphorylation of p65 serine 536 might be the underlying molecular mechanism for the NF-κB inhibition [13].

The role of TBK1 in nociception has not been investigated so far although several data hint at important TBK1 functions, particularly in inflammatory processes. TBK1 modulation has been associated with regulation of inflammatory mediators such as IL-6, TNF-α, or IFN-β [14, 15], and TBK1-deficient cells show strong defects in IFN induction [16]. TBK1-depleted fibroblast-like synoviocytes revealed reduced capacity for IRF-3-mediated induction of IFN-β and IP-10, suggesting that, particularly, TBK1 is involved in the pathogenesis of rheumatoid arthritis [17]. Furthermore, TBK1 is involved in insulin receptor signaling and might therefore form a link between inflammation and insulin resistance/diabetes [18]. In this study, we investigated its expression, regulation, and function in the nervous system during inflammatory nociception in two mouse models of inflammatory hyperalgesia to determine the impact of TBK1 and its potential downstream target genes on pathophysiological pain.

## Materials and methods

### Animals

C57BL/6 mice were obtained from Harlan Winkelmann, Germany at the age of 6–8 weeks. TBK1^−/−^/TNFR^−/−^-mice were kindly provided by Prof. Shizuo Akira, Japan. For TBK1 deletion, a 1.5-kbp fragment in exon 9 of the murine *TBK1* gene was replaced with a neomycin-resistant gene cassette. Mice heterozygous for TBK1 remain alive and healthy. However, TBK1^−/−^ mice have been shown to die around embryonic day 14.5 (E14.5) [16]. Therefore, mice have to be bred on a TNF-receptor knock-out (TNFR^−/−^) background [19] which prevents premature death [20]. TBK1^−/−^/TNFR^−/−^ mice were crossed with TBK1^+/+^/TNFR^−/−^ mice and heterozygous offspring were then mated to generate TBK1^+/+^/TNFR^−/−^ and TBK1^−/−^/TNFR^−/−^ littermates. Backcrossing with C57BL/6 wild type mice was performed to generate TBK1^+/+^/TNFR^+/+^ littermates. Genotyping was performed using the primers in Table 1.

**Table 1:** Primer sequences for genotyping

	Primers
TBK1 wild	5′- CTAATGGTTGTAGTCAGGGTCTCCTGC -3′
TBK1 extra	5′- TGCGTTCCTGTCCTGACCGTGATTGTG -3′
TBK1 neo	5′- ATCGCCTTCTATCGCCTTCTTGACGAG -3′
TNFRSense1	5′- AGATGGAGAAGGGCAGTTAG -3′
AntiS1	5′- ATACCAGGGTTTGAGCTCAG -3′
AntiS2	5′- TACTTTGTTAAGAAGGGTGAGA -3′

For experiments, we used 2–3-month-old littermate mice. Animals had free access to food and water and were maintained in climate- and light-controlled rooms (24 ± 0.5 °C, 12/12 dark/light cycle). In all experiments, the ethical guidelines for investigations in conscious animals were obeyed and the procedures were approved by the local Ethics Committee for Animal Research (F95/53). All efforts were made to minimize animal suffering and to reduce the number of animals used. All behavioral experiments were assessed by a blinded observer in a dedicated room with restricted sound level and activity.

### Western blot analysis

For Western blot analysis, mice were injected with either formalin or zymosan A into the hind paws. Lumbar spinal cords (L4-L6) were dissected out at the indicated time points. Tissues were homogenized in PhosphoSafe Extraction Buffer (Merck, Darmstadt, Germany) with protease inhibitor mixture (1 mM Pefabloc SC Alexis Biochemicals, Lausen, Switzerland) immediately after preparation. To remove cellular debris, extracts were centrifuged at 16.800 × *g* for 1 h at 4 °C. The supernatants were stored at –80 °C. Proteins from tissue homogenates (15–30 μg) were separated electrophoretically by 10 % SDS-PAGE and then transferred onto nitrocellulose membranes by wet-blotting. To confirm equal loading all blots were stained with Ponceau S red solution. Membranes were blocked for 60 min at room temperature in Odyssey blocking reagent (LI-COR Biosciences, Bad Homburg, Germany) diluted 1:2 in 0.1 M PBS, pH 7.4. Then the blots were incubated overnight at 4 °C with primary antibody against TBK1 (84 kDa), p42 (42 kDa), p-p42 (42 kDa), p44 (44 kDa), p-p44 (44 kDa), JNK (46 kDa), p-JNK (46 kDa), SAPK (54 kDa), p-SAPK (54 kDa), p-p65(Ser536) (65 kDa) (all 1:250, Cell Signaling Technology, Boston, USA) in blocking buffer. After washing three times with 0.1 % Tween 20 in 0.1 M PBS, the blots were incubated for 60 min with an IRDye 800- or IRDye 700-conjugated secondary antibody (1:5000 in blocking buffer). After rinsing in 0.1 % Tween 20 in 0.1 M PBS, protein-antibody complexes were detected with the Odyssey Infrared Imaging System (LI-COR, Bad Homburg, Germany). β-Actin (37 kDa), (1:1200, Sigma) or Hsp90 (90 kDa), (1:500, BD Bioscience) was used as loading control. Densitometric analysis of the blots was performed with Image Studio Lite Software (LI-COR, Bad Homburg, Germany).

### Real-time PCR (Taqman)

RNA was prepared from the lumbar spinal cords and dorsal root ganglia (DRGs) (L4-L6) using TRI reagent according to the standard procedure [13]. Two hundred nanogram of total RNA was used for the reverse transcription which was performed with Random and Oligo-dT Primers (2:1 ratio) in a Verso cDNA Synthesis Kit (Thermo Scientific, Darmstadt, Germany). Twenty nanogram RNA equivalent was subjected to real-time PCR in an Applied Biosystems sequence detection system AB7500 using the FastStart Universal SYBR Green Master (Rox) System (Roche Diagnostics, Mannheim, Germany). Expression of TBK1, COX-2, iNOS, MMP-9, c-fos, and Akt1 mRNA was assessed related to GAPDH mRNA. The following gene-specific primers were used:

1. FW5′-GAG CCG GGA ACA ACT CAA TA-3′
  RV5′-TTG CTT TTG TGG CAT GGT AA-3′
2. FW5′-AGACACTCAGGTAGACATGATCTACCCT-3′
  RV5′-GGCACCAGACCAAAGACTTCC-3′
3. FW5′-TCACCCACACTGTGCCCATCTACGA-3′
  RV5′-CAGCGGAACCGCTCATTGCCAATGG-3′
4. FW5′-GAAGCCAAACCCTGTGTGTT-3′
  RV5′-AGAGTACTGCTTGCCCAGGA-3′
5. FW5′-ACCATGATGTTCTCGGGTTTCAA-3′
  RV5′-GCTGGTGGAGATGGCTGTCAC-3′
6. FW5′-GGCTGGCTGCACAAACG-3′
  RV5′-GACTCTCGCTGATCCACATCCT-3′
7. FW5′-CAA TGT GTC CGT CGT GGA TCT-3′
  RV5′-GTC CTC AGT GTA GCC CAA GAT G-3′

The cycle number at which the fluorescence signal crosses a defined threshold (Ct-value) is proportional to the number of RNA copies present at the start of the PCR. The threshold cycle number for the specific mRNA was standardized by subtracting the Ct-value of GAPDH from the Ct-value of gene-specific amplifications of the same sample. Relative quantitative level of samples was determined by standard 2^(-ΔΔCt)^ calculations and expressed as fold-change in a single reference control sample (untreated control mice of either genotype).

### Immunofluorescence

Mice were perfused intracardially with 0.9 % saline followed by 4 % paraformaldehyde in 0.1 M phosphate-buffered saline (PBS), pH 7.4, under deep ketamine/xylazine anesthesia. Lumbar spinal cords (L4-L6) were dissected out, post-fixed in 4 % PFA in 0.1 M PBS (pH 7.4), cryoprotected in 20 % sucrose in 0.1 M PBS overnight at 4 °C and then embedded in Tissue-Tek O.C.T. Compound (Sakura Finetek Europe B.V., Alphen aan den Rijn, Netherlands) frozen on dry ice. Cryostat sections were cut at a thickness of 16 μm and stored at −80 °C. For immunofluorescence, slices were permeabilized for 15 min with 0.1 M PBS containing 0.1 % Triton-X 100. The sections were then blocked in 3 % BSA in 0.1 M PBS for 1 h to reduce non-specific binding. After rinsing in 0.1 M PBS, sections were incubated with TBK1 antibody (1:500, Acris) and for co-immunofluorescence with mouse anti-neuronal nuclei (NeuN, 1:1000, Sigma Aldrich), mouse anti-GFAP (1:1000, Chemicon), or CD11b (1:500). Antibodies were dissolved in 0.1 % Tween 20 in 0.1 M PBS and incubated at 4 °C overnight. After rinsing in 0.1 M PBS, sections were incubated for 2 h at room temperature with Alexa Flour 488- or Cy3-conjugated secondary antibodies dissolved in 0.1 % Tween 20 in 0.1 M PBS, rinsed again in 0.1 M PBS and coverslipped with Fluoromount G. Images were captured using an inverted fluorescence microscope (Axio Observer.Z1; Zeiss, Germany) equipped with a monochrome CCD camera and AxioVision software (Zeiss, Germany).

### Transcription factor analysis

Nuclear extracts from the spinal cord or cell culture were prepared using a commercially available Nuclear Extract Kit following the manufacturer’s instructions (Active Motif, Rixensart, Belgium). These nuclear extracts were then subjected to TransAM^TM^ NF-κB p65, c-fos, and IRF-3 transcription factor ELISAs (Active Motif). Five microgram nuclear protein was allowed to bind to an oligonucleotide-coated plate. NF-κB p65, c-fos, or IRF-3 was detected by incubation with specific primary antibodies and HRP-conjugated secondary antibody. The colorimetric readout was measured photometrically at 450 nm and is proportional to transcription factor activity.

### Behavioral testing

Littermates were used in all behavioral tests. Animals were habituated to the experimental room and were investigated by observers blinded for the genotype of the animals. Genotyping was done as described above.

#### Rotarod test

Motor coordination was assessed with a Rotarod Treadmill for mice (Ugo Basile, Comerio, Italy) at a constant rotating speed of 32 rpm. All mice had five training sessions before the day of the experiment. The fall-off latency was averaged from five tests. The cut-off time was 90 s.

#### Mechanical sensitivity

Paw withdrawal latency in response to mechanical stimulation was assessed with an automated testing device consisting of a steel rod that is pushed against the plantar surface of the paw with increasing force until the paw is withdrawn (Dynamic Plantar Aesthesiometer, Ugo Basile, Varese, Italy). The maximum force was set at 5 g to prevent tissue damage and the ramp speed was 0.5 g/s. The cut-off time was 20 s. Mice were placed in test cages with a metal grid bottom. After 1-h accommodation time, the paw withdrawal latency was measured. The mean of 4–6 consecutive trials at each time point were used for further analysis.

#### Hot-plate test

Animals were placed into a Plexiglas cylinder on a heated plate maintained at 52.0 ± 0.2 °C (Ugo Basile), and the latency to jump, shake/flutter a hind paw, or licking the front paws was recorded. Each animal was tested only once since repeated testing in this assay leads to latency changes [21]. The cut-off time was 30 s.

#### Formalin test

The formalin test was performed as described [22]. Mice were placed on a table top within a Plexiglas cylinder and were allowed to habituate for 30 min. Twenty microliters of a 5 % formaldehyde solution (formalin) was injected subcutaneously into the dorsal surface of the left hind paw. The time spent licking the formalin-injected paw was recorded at 5 min intervals up to 45 min, starting right after formalin injection. Two hours after, formalin injection animals were deeply anesthetized with isoflurane and then killed by cardiac puncture. Lumbar spinal cords and DRGs (L4-L6) were dissected out and immediately frozen in liquid nitrogen and stored at −80 °C until further preparation.

#### Zymosan A-induced paw inflammation

Paw withdrawal latency in response to mechanical stimulation was assessed as described above. Mice were placed in test cages with a metal grid bottom. After 1 h, baseline paw withdrawal latencies were measured. On the next day, hind paw inflammation was induced by subcutaneous injection of 20 μl of a 10 mg/ml zymosan A (Sigma-Aldrich, Munich, Germany) suspension in phosphate-buffered saline (0.1 M, pH 7.4) into the mid plantar region of the left hind paw [23]. The mean of four consecutive trials with 10 s intervals was determined hourly up to 8 h after zymosan A injection and then again after 24 and 48 h. At the end of the observation period (48 h after zymosan A injection), animals were deeply anesthetized with isoflurane and then killed by cardiac puncture. Lumbar spinal cords and DRGs (L4-L6) were dissected out and immediately frozen in liquid nitrogen and stored at −80 °C until further preparation. Ipsi- and contralateral paws were detached at the ankle. The paw volume was determined by weighing the paws.

### Cell culture

RAW264.7 mouse macrophages were cultured and incubated in RPMI 1640 medium containing 10 % FCS and 1 % penicillin/streptomycin. Cells were stably transduced with either TBK1-specific shRNA (GIPZ-system, Thermo Scientific, Germany) or negative control virus (NC) and cultivated under puromycin selection. Permanent expression of shRNA resulted in 80 % reduction of the TBK1 protein level, which remained stable throughout the whole incubation period (Additional file 1: Figure S6 A). Bone marrow derived macrophages (BMM) were generated from TNFR^−/−^ and TBK1^−/−^/TNFR^−/−^ mice. Muscle tissue was removed from the hind legs, and the bones were cut directly beyond and above the joints. The bones were placed into a perforated 0.2 ml tube, which was inserted into a 1.5 ml reaction tube. After centrifugation at 16.800 × *g* for 15 s, the bone marrow was resuspended in RPMI 1640 medium supplemented with 10 % FCS, 1 % Pen/Strep, and 20 ng/ml recombinant murine macrophage colony stimulating factor (M-CSF, Peprotech, Hamburg, Germany). Cells were seeded in six well plates and cultivated and differentiated for 7 days before stimulation. RAW264.7 and BMM were stimulated with 10 μg/ml lipopolysaccharide (LPS) for the indicated time points. Protein was isolated using Phophosafe/Pefabloc (Merck/Novagen, Darmstadt, Germany/Alexis Biochemicals, Lörrach, Germany) according to manufacturer’s instruction.

### Data analysis

Statistical evaluation was done with Graph Pad Prism 5.0 for Windows. Data are presented as mean ± SEM. Data were either compared by univariate analysis of variance (ANOVA) with subsequent *t* tests employing a Bonferroni α-correction for multiple comparisons or by student’s *t* test. For analysis of inflammatory hyperalgesia in the zymosan A-induced paw inflammation, paw withdrawal latencies in response to mechanical stimulation were expressed as the relative difference between the zymosan A-treated left and the untreated right hind paw calculated as ΔPWL = (left-right) / right × 100 (mean ± SEM). For analysis of inflammatory hyperalgesia in the zymosan A-induced paw inflammation and the formalin test, repeated measures ANOVA was performed. For all tests, a probability value *P* < 0.05 was considered as statistically significant.

## Results

### Expression and regulation of TBK1 in the spinal cord and the DRGs

**Fig. 1:**
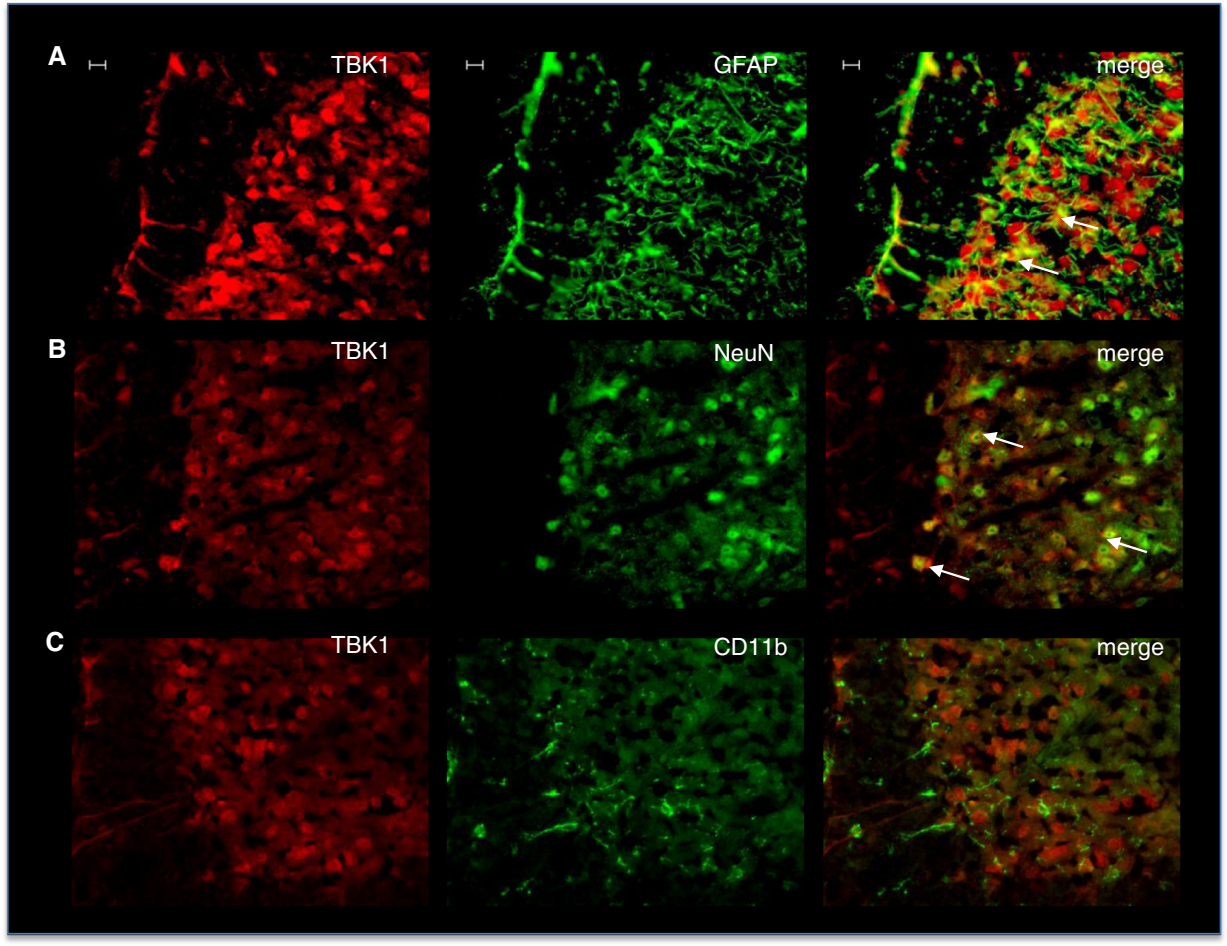
TBK1 expression in the dorsal spinal cord. Representative co-immunofluorescence showing TBK1 protein expression in the dorsal horn of spinal cord in combination with cell markers for (**a**) astrocytes (GFAP), (**b**) neurons (NeuN), and (**c**) microglia (CD11b). TBK1 was stained with Cy3 (*red*), cell markers with Alexa Fluor 488 (*green*). The three images show from left to right: TBK1 alone, cell marker alone, and merged (representative result from three independent experiments). The arrows indicate double-labeled cells. Scale Bar, 10 μm

**Fig. 2:**
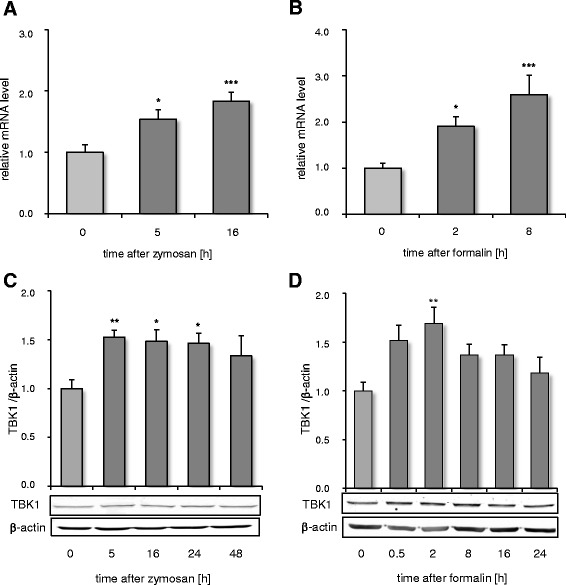
TBK1 regulation in the spinal cord after peripheral inflammatory stimulation. Time course of the TBK1 mRNA and protein expression in the lumbar spinal cord after peripheral injection of zymosan A (**a**, **c**) and formalin (**b**, **d**), respectively (*n* = 4 mice/group). The Western blots (**c**, **d**) show one representative blot; the diagrams depict the densitometric analysis of all blots (mean ± S.E.M). Univariate ANOVA with Bonferroni post-hoc analysis *P < 0.05, **P < 0.01, and ***P < 0.001

Constitutive TBK1 expression in the spinal cord was detected by immunofluorescence (Fig. 1). The specificity of the TBK1 antibody was confirmed with tissues from TBK1^−/−^/TNFR^−/−^ mice, which showed no signal (Additional file 2: Figure S1). Co-staining of naïve wild type tissues with markers for neurons, microglia, and astrocytes revealed TBK1 localisation in NeuN-positive neurons and some GFAP-positive astrocytes. CD11b-positive microglial cells showed no co-staining with TBK1 indicating that it is not expressed in this cell type under naïve conditions (Fig. 1). Accordingly, we found low levels of basal TBK1 expression in primary microglia and astrocytes, which however were upregulated upon inflammatory stimulation with a cytokine mix (Additional file 3: Figure S2). To gain an impression whether TBK1 expression in the spinal cord is due to localization in neurons of the terminals of primary nociceptive afferents, immunofluorescence images from DRG slides were prepared additionally. TBK1 expression was detectable in small non-myelinated nociceptive neurons and large myelinated non-nociceptive neurons of the DRGs, as assessed by co-staining with IB4 and Neurofilament 200, respectively (Additional file 4: Figure S3), indicating its role in nociceptive transmission as well as general functions in sensory perception in the periphery. Potential regulation of spinal TBK1 during nociception was assessed in two different inflammatory models in mice: the formalin test which reflects short time tonic nociception associated with mechanisms of central sensitization and the zymosan A-induced paw inflammation model which leads to the formation of paw edema and increased sensitivity to nociceptive stimuli that lasts for several days. In both models, we observed an increase in TBK1 mRNA and protein levels in the spinal cord after peripheral noxious stimulation (Fig. 2) suggesting that TBK1 is a modulator of inflammatory nociception and sensitization towards ongoing nociceptive input. In contrast, no TBK1 regulation after inflammatory nociceptive stimulation could be observed in DRGs indicating its main function in nociceptive processing in the central nervous system (Additional file 4: Figure S3).

### TBK1 knock-out is associated with antinociceptive effects in inflammatory models

Genetically modified mice were used to further evaluate TBK1 function in nociceptive processing. Before starting nociceptive tests, the Rotarod Test was performed to assess intact motoric coordination of TBK1^−/−^/TNFR^−/−^ and TNFR^−/−^ mice, which is an important prerequisite for proper behavioral nociceptive testing. Neither TBK1^−/−^/TNFR^−/−^ nor TNFR^−/−^ mice showed any differences in their ability to balance on the rotating rod and remained there until the cut-off time of 90 s indicating that the genetic modification does not impair their motor functions. Hot-plate and Dynamic Plantar tests were applied to assess the impact of the genetic modifications on acute thermal and mechanical nociception, respectively. The results of both tests showed that there were no significant differences between TBK1^−/−^/TNFR^−/−^ and TNFR^−/−^ mice indicating that the physiologically important immediate response to acute noxious thermal and mechanical stimulation is unaffected in the knock-out mice (mean ± SEM; Hot Plate: TNFR^−/−^ 14.7 ± 1.1; TBK1^−/−^/TNFR^−/−^ 19.4 ± 2.0 (*P* = 0.101); Dynamic Plantar: TNFR^−/−^ 7.7 ± 0.6; TBK1^−/−^/TNFR^−/−^ 7.6 ± 0.6). Due to the homology of TBK1 with other IκB kinases and a potential compensatory regulation of similar proteins in response to systemic knock-out of genes, we determined the protein levels of IKKα, IKKβ, and IKKε in spinal cord protein extracts of TBK1^−/−^/TNFR^−/−^, TNFR^−/−^, and wild type mice. The results of the Western blot analysis showed equal protein expression for all kinases in all genotypes tested suggesting that these proteins were not compensatory regulated in TBK1 knock-out mice (data not shown).

**Fig. 3:**
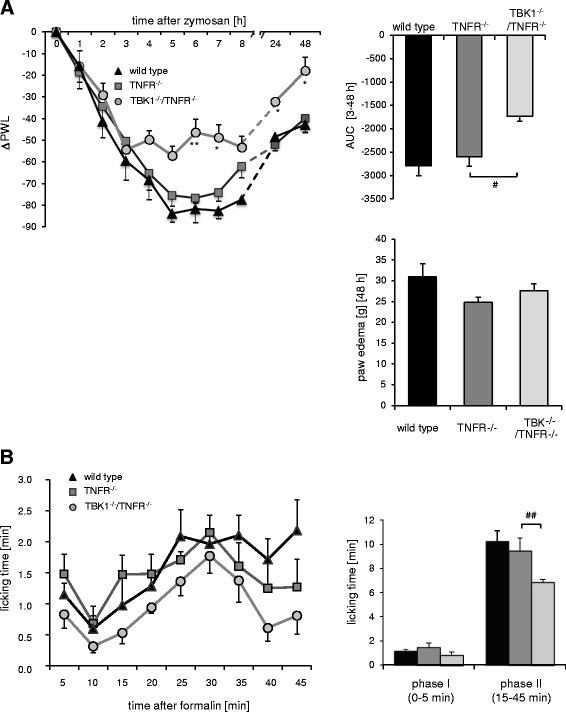
Inflammatory hyperalgesia in TNFR^−/−^ and TBK1^−/−^/TNFR^−/−^ mice. **a** Left panel: time course of mechanical hyperalgesia in wild type (*black triangle*), TNFR^−/−^ (*dark grey square*), and TBK1^−/−^/TNFR^−/−^ (*light grey circle*) mice after injection of 10 mg/ml (20 μl) zymosan A into a hind paw. The diagram shows the delta paw withdrawal latencies (ΔPWL) in response to mechanical stimulation as assessed with a Dynamic Plantar Aesthesiometer (*n* = 6 mice/group), repeated measure ANOVA with Bonferroni post-hoc analysis, *P < 0.05 and **P < 0.01. Upper right panel: comparison of the area under the paw withdrawal latency versus time curve between wild type (black column), TNFR^−/−^ (*dark grey column*), and TBK1^−/−^/TNFR^−/−^ (*light grey column*) mice 3 to 48 h after zymosan A injection. Univariate ANOVA with Bonferroni post-hoc analysis ^#^ *P* < 0.05 significant mean difference between the respective genotypes. Lower right panel: determination of the paw weight of wild type (black column), TNFR^−/−^ (*dark grey column*), and TBK1^−/−^/TNFR^−/−^ (*light grey column*) mice 48 h after zymosan A injection. **b** Left panel: time course of the licking behavior in wild type (*black triangle*), TNFR^−/−^ (*dark grey square*), and TBK1^−/−^/TNFR^−/−^ (*light grey circle*) mice (*n* = 8 mice/group) after injection of formalin (5 %, 20 μl) into the hind paw. Formalin was injected at time ‘0’, and the time spent licking the injected paw was measured at 5 min intervals for 45 min. Right panel: statistical analysis of phase I (0–5 min) and phase II (15–45 min) between wild type (black column), TNFR^−/−^ (*dark grey column*), and TBK1^−/−^/TNFR^−/−^ (*light grey column*). Univariate ANOVA with Bonferroni post-hoc analysis ^##^ *P* < 0.01 significant mean difference between the respective genotypes

In the inflammatory nociceptive models, wild type littermates showed typical nociceptive responses. After injection of zymosan A, a strong decrease in paw withdrawal latencies towards mechanical stimulation was observed which lasted until the end of the experiment at 48 h (Fig. 3a). This effect was already reduced in TNFR^−/−^ animals and further alleviated in TBK1^−/−^/TNFR^−/−^ mice in the period from 3 h up to 48 h after zymosan A injection. Latencies almost returned to baseline in TBK1^−/−^/TNFR^−/−^ mice at 48 h (repeated measures ANOVA, *P < 0.05 (TNFR^−/−^) and **P < 0.01 (TBK1^−/−^/TNFR^−/−^). To investigate the impact of TBK1 on paw inflammation, the zymosan A-injected paws were weighted at the end of the experiments to determine the edema volume. Since no significant differences were observed between the genotypes (Fig. 3a), it has to be suggested that TBK1 does not directly influence the inflammatory response in the paw. This assumption was further supported by RT-PCR analyses of paw tissue. Although TBK1 in wild type mice and the inflammatory cytokines IL-1β, IL-6, and IFN-β1 in wild type, TBK1^−/−^/TNFR^−/−^ and TNFR^−/−^ animals were strongly upregulated after inflammatory stimulation, there were no significant differences in the level of inflammatory cytokines between the genotypes (Additional file 5: Figure S4).

Following injection of formalin, TNFR^−/−^ as well as TBK1^−/−^/TNFR^−/−^ mice showed the typical biphasic nociceptive behavior consisting of licking the injected paw. The first phase (0–5 min) is due to chemical-fiber stimulation of peripheral nociceptors [24]. After a gap phase of ~10 min, the second phase lasts from 15–45 min and corresponds to spinal sensitization. In comparison to wild type mice, we already found an impaired nociceptive response in mice with a deletion of TNFR. However, TBK1^−/−^/TNFR^−/−^ mice revealed a further decrease in licking of the formalin-injected hind paw which was significant in the second phase of the formalin assay as compared to TNFR^−/−^ mice (Fig. 3b) indicating that TBK1 makes an important contribution to the formalin-induced C-fiber sensitization.

### Regulation of NF-κB and IRF3 activity

**Fig. 4:**
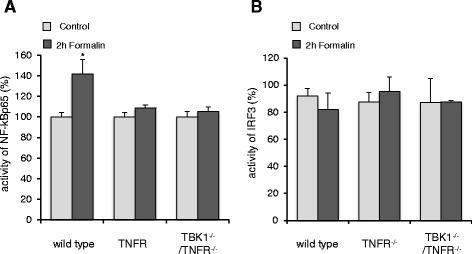
Regulation of NF-κB and IRF3 activation in the spinal cord. **a** p65 and **b** IRF3 transcription factor activity as assessed by TransAM transcription factor ELISA. Nuclear extracts of the spinal cord of wild type, TNFR^−/−^ and TBK1^−/−^/TNFR^−/−^ mice were prepared from control mice and mice 2 h after injection of formalin into the hind paws (*n* = 3 mice/group); bright columns = control; dark columns = 2 h formalin, *P < 0.05, statistically significant differences compared with untreated control

**Fig. 5:**
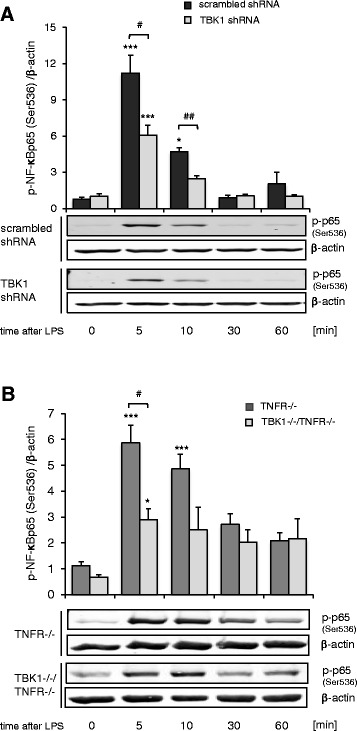
Regulation of NF-κB in RAW264.7 and primary bone marrow macrophages. **a** Western blot showing serine 536 phosphorylation of p65 in LPS-stimulated RAW264.7 macrophages stably transduced with scrambled (*black columns*) or TBK1-specific shRNA (*light grey columns*), (*n* = 4). The blots show a representative result; the diagram shows the densitometric analysis of all experiments. **b** Western blot showing serine 536 phosphorylation of p65 in LPS-stimulated BMMs derived from TNFR^−/−^ (*dark grey columns*) and TBK1^−/−^/TNFR^−/−^ (*light grey columns*) mice, (*n* = 4). The blots show a representative result, the diagram shows the densitometric analysis of all experiments. Univariate ANOVA with Bonferroni post-hoc analysis *P < 0.05 and ***P < 0.001 statistically significant differences compared with unstimulated cells. Student’s *t* test ^#^ *P* < 0.05 and ^##^ *P* < 0.01 significant mean difference between scrambled shRNA and TBK1 shRNA or TNFR^−/−^ and TBK1^−/−^/TNFR^−/−^ cells, respectively

Since NF-κB as well as IRF3 are important targets of TBK1, we studied the regulation of these transcription factors in the spinal cord of wild type, TBK1^−/−^/TNFR^−/−^ as well as TNFR^−/−^ mice with and without formalin stimulation (Fig. 4). NF-κB was significantly activated in nuclear extracts of the spinal cord of wild type animals after peripheral formalin injection while IRF3 activity remained unaltered. In accordance with these data, IRF3 was neither affected in TBK1^−/−^/TNFR^−/−^ nor in TNFR^−/−^ mice in this study. However, in contrast to the activation of NF-κB in wild type mice, we found no significant effect of formalin in either TBK1^−/−^/TNFR^−/−^ or TNFR^−/−^ mice indicating that the knock-out of TNFR already inhibits NF-κB activation which covers possible effects of TBK1. Therefore, cell culture experiments with RAW264.7 mouse macrophages, which were stably transduced with TBK1 shRNA were additionally performed. In comparison to control cells, which showed a significant increase in phosphorylated NF-κB p65 (Ser536) 5 and 10 min after stimulation with LPS, knock-down of TBK1 by shRNA significantly decreased this phosphorylation by about 50 % (Fig. 5a). These results are further supported by experiments with primary bone marrow macrophages (BMM) derived from TBK1^−/−^/TNFR^−/−^ and TNFR^−/−^ mice. The LPS-induced increase of p65 (Ser536)-phosphorylation after 5 and 10 min stimulation in TNFR^−/−^ BMMs was significantly reduced in BMMs from TBK1^−/−^/TNFR^−/−^ mice (Fig. 5b) indicating that TBK1 is involved in stimulus-dependent NF-κB activation which might at least partially contribute to the effects observed in the nociceptive animal models.

### Regulation of potential TBK1 downstream targets

**Fig. 6:**
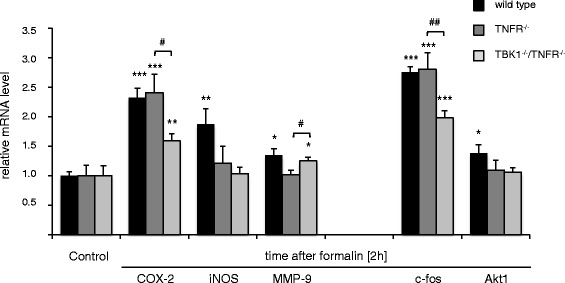
Regulation of NF-κB dependent and independent genes in the spinal cord. Quantitative RT-PCR (Taqman) of cyclooxygenase-2 (COX-2), inducible NO-synthase (iNOS), matrix metalloproteinase-9 (MMP-9), c-fos, and Akt1 mRNA in the spinal cord of naïve mice and 2 h after formalin injection into a hind paw. GAPDH-RNA was used as reference control gene (*n* = 4 mice/group) and each Taqman-PCR was performed twice in triplicate; black columns = wild type; dark grey columns = TNFR^−/−^, light grey columns = TBK1^−/−^/TNFR^−/−^, Student’s *t* test *P < 0.05, **P < 0.01, and ***P < 0.001 in comparison to untreated controls, ^#^ *P* < 0.05, ^##^ *P* < 0.01 significant mean difference between TNFR^−/−^ and TBK1^−/−^/TNFR^−/−^ mice

**Fig. 7:**
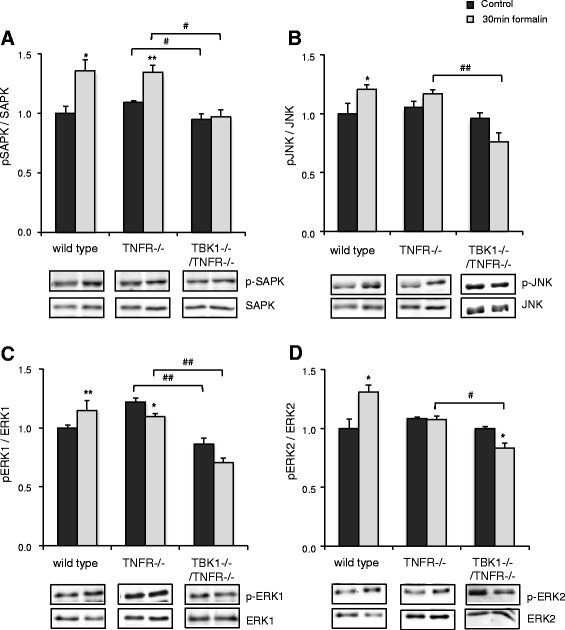
Regulation of MAPK activity in the spinal cord of wild type, TNFR^−/−^ and TBK1^−/−^/TNFR^−/−^ mice. Western blots showing the activation of SAPK (**a**), JNK (**b**), ERK1 (**c**), and ERK 2 (**d**) in different genotypes as assessed by Western blot analysis with phospho-specific antibodies. The blots were normalized to the respective total protein levels and additionally against the loading control HSP90 or β-actin. The blots show a representative result, the diagram shows the densitometric analysis of three independent experiments (*n* = 3 mice/group); dark columns = control; bright columns = 30 min formalin. Student’s *t* test *P < 0.05 and **P < 0.01 significant mean difference between untreated and formalin–injected mice. ^#^ *P* < 0.05 and ^##^ *P* < 0.01 significant mean difference between TNFR^−/−^ and TBK1^−/−^/TNFR^−/−^ mice

Further experiments were performed to clarify which signal transduction pathways participate in the TBK1-mediated nociception in the spinal cord. Since NF-κB activation was only partially inhibited by downregulation of TBK1 in RAW264.7 macrophages or BMMs from TBK1^−/−^/TNFR^−/−^ mice, we suggested that TBK1 downstream targets are not limited to the NF-κB cascade and analyzed a panel of NF-κB dependent as well as independent “pain-relevant” genes. The NF-κB dependent genes cyclooxygenase-2 (COX-2), inducible nitric oxide synthase (iNOS), and matrix metalloproteinase-9 (MMP-9) showed the expected upregulation in the spinal cord after peripheral formalin injection in wild type mice. iNOS and MMP-9 induction were repressed in both TBK1^−/−^/TNFR^−/−^ and TNFR^−/−^ mice in a similar manner, whereas COX-2 regulation was normal in TNFR^−/−^ mice and significantly decreased in TBK1^−/−^/TNFR^−/−^ animals. For the NF-κB-independent genes c-fos and Akt1, a formalin-induced increase in the spinal cord was observed in wild type mice. The upregulation of Akt1 was inhibited in both TBK1^−/−^/TNFR^−/−^ and TNFR^−/−^ mice, whereas c-fos modulation was significantly repressed in TBK1^−/−^/TNFR^−/−^ animals but remained unaltered in TNFR^−/−^ mice (Fig. 6). These data indicate that COX-2 and c-fos strongly contribute to the TBK1-mediated nociception. The role of COX-2 in TBK1 signaling in nociception was further confirmed by experiments with the COX-2 selective inhibitor celecoxib. While celecoxib treatment was able to further reduce the antinociceptive effects of the TNFR-deletion in a significant manner, the reduction in TBK1^−/−^/TNFR^−/−^ mice was only marginal thus further supporting the hypothesis that COX-2 is a TBK1 downstream target (Additional file 6: Figure S5). The strong regulation of c-fos prompted us to investigate the regulation of MAP kinases which are involved in the control of c-fos expression [25, 26] and have been repeatedly associated with nociceptive responses [27–29]. As expected, we found a significant increase in ERK 1/2, SAPK, and JNK activity in the spinal cord of wild type mice after injection of formalin into the paw as assessed by Western blot with phospho-specific antibodies. The formalin-induced phosphorylation of ERK 1/2 and JNK was already inhibited in TNFR^−/−^ mice, but in TBK1^−/−^/TNFR^−/−^ mice, we even found a decrease in their phosphorylation to below baseline level. The increase in p-SAPK levels was similar in wild type and TNFR^−/−^ mice but significantly inhibited in TBK1^−/−^/TNFR^−/−^ mice (Fig. 7). These results indicate that TBK1 contributes to c-fos activation in inflammatory nociception by regulation of MAP kinase activation. This is supported by cell culture experiments with TBK1 depleted RAW264.7 macrophages which showed a 50 % reduced c-fos activation in comparison to control cells in which activated c-fos was strongly increased 60 min after LPS stimulation (Additional file 1: Figure S6 B).

## Discussion

The classical I-κB kinase complex consisting of the two catalytical subunits IKKα and β and the regulatory subunit IKKγ (NEMO) has been suggested to be an essential player in inflammatory NF-κB activation [30] and also contributes to inflammatory nociception [10, 31]. In a recent study, we found that the IKK-related IKKε is also involved in processing of inflammatory pain by regulating NF-κB activity and the expression of NF-κB dependent genes [13]. TBK1 constitutes another non-canonical IKK which might form a complex with IKKε and is involved in regulation of antiviral responses by modulating IFN Type I and also in inflammatory processes by regulating NF-κB signaling (reviewed in [7, 32, 33]). The aim of this study was to clarify whether TBK1 is also involved in inflammatory nociceptive processing. We observed TBK1 expression in “pain-relevant” cells in the spinal cord and the DRGs. After peripheral inflammatory nociceptive stimulation, TBK1 was upregulated in the spinal cord but not in the DRGs indicating its main role in the central nervous system. Effects in the formalin model could be detected at earlier time points compared to the zymosan A model which might reflect the fact that the formalin model constitutes a more acute inflammatory nociceptive model leading to early effects while zymosan A-induced paw inflammation provides more information about persistent hyperalgesia and later regulation [23]. TBK1 knock-out mice were used to assess inflammatory hyperalgesia in the two different mouse models after confirming that similar kinases such as IKKα, β, and ε do not compensate for the TBK1 deletion. Due to embryonic lethality of complete TBK1 knock-out mice as a result of liver degeneration and apoptosis [16, 34], the TBK1^−/−^ mice had to be bred under a TNFα-receptor knock-out and were compared to TNF-α receptor knock-out mice (TNFR^−/−^) as controls. A knock-out of TNFR in mice has already been repeatedly associated with reduced nociceptive responses in models of inflammatory, cancer, and neuropathic pain [35–37] and might thus cover antinociceptive effects mediated by TBK1 knock-down. Accordingly, we showed that the TNFR knock-out already ameliorates nociception in the formalin test and in the zymosan A-induced paw inflammation. Nevertheless, in TBK1/TNFR double-knock-out mice, this effect was significantly enhanced indicating that TBK1 contributes to inflammatory hyperalgesia. These results are in accordance with studies showing anti-inflammatory effects of TBK1 inhibition in fibroblast-like synoviocytes by reducing the expression of the proinflammatory protein IP-10 [17]. Furthermore, TBK1 is involved in the inflammatory response in obesity and hypertension and contributes to phosphorylation of the insulin receptor thus impairing its function and supporting insulin resistance [18]. Treatment of obese mice with the TBK1/IKKε inhibitor amlexanox led to weight loss and improved insulin sensitivity by elevation of energy expenditure [38, 39], which further confirms that TBK1 contributes to proinflammatory reactions. Our concern was to investigate whether TBK1 effects in nociception are mediated via NF-κB signaling possibly in concert with IKKε or via other pathways. In a recent study, we used BX795 as a pharmacological inhibitor of IKKε and TBK1 and showed that the combined inhibition of both kinases led to a stronger reduction of the noxious response in comparison to single IKKε knock-out [13]. Together, with the results of this study, it seems that inhibition of both kinases by BX795 leads to an addition of the effects of IKKε- and TBK1-mediated analgesia which might be due to synergistic modulation of the same target genes or to regulation of different targets, respectively. For IKKε, we showed that a complete inhibition of NF-κB p65 phosphorylation at Ser 536 was responsible for inhibition of NF-κB dependent target genes, such as COX-2, iNOS, and MMP-9, while NF-κB independent targets were not affected [13]. The results of this study revealed that a knock-down of TBK1 also inhibits NF-κB activation by decreasing phosphorylation of p65 Ser536 but to a lesser extent than that shown for IKKε inhibition. From these data and published studies which hint towards divergent functional roles for TBK1 and IKKε [5, 6], we postulated that TBK1 might affect other target genes in addition. Indeed, we found that the NF-κB-independent marker of neural activity, c-fos, was also differentially regulated in TBK1 knock-out mice in comparison to TNFR^−/−^ and wild type mice in association with a decreased activation of the MAP kinases ERK1/2 and SAPK/JNK. These MAPKs have been frequently associated with nociceptive responses [27–29] and inhibition of their activation might contribute to antinociceptive effects in TBK1 knock-out mice. A relationship between TBK1 and MAPKs has already been found in a study with TBK1^−/−^ mouse embryonic fibroblasts (MEFs) which showed reduced TNFα-induced MAPK activation in comparison to TBK1^+/+^ cells [40]. In contrast, TBK1 was not involved in MAPK activation stimulated by double stranded DNA [41] or TLR-3 activation [42]. A recent study has further shown that NF-κB activation is capable of participation in c-fos induction, but only in concert with ERK-mediated fos induction [43], a mechanism which might also have contributed to TBK1 signaling in this study and supports the increase in c-fos. IRF3, which has been frequently described as a prominent TBK1 target, seems to play only a minor role in nociception, since it was not affected by nociceptive stimulation or by deletion of TBK1.

## Conclusions

This study shows that TBK1, similar to IKKε, is involved in inflammatory nociceptive processing in the spinal cord and might therefore constitute a promising target for pharmacological intervention in pain. Our data indicate that, on the one hand, TBK1 and IKKε work together in a complex to inhibit NF-κB signal transduction while, on the other hand, TBK1 additionally exerts IKKε-independent actions by the so far undescribed in vivo regulation of MAP kinases and c-fos. Nevertheless, the embryonic lethality of a complete TBK1 knock-out raises concerns that inhibition of TBK1 in humans would be associated with severe unwanted side effects. A possible solution to this problem might be tissue-specific modulation of TBK1 or drugs with combined IKKε/TBK1 inhibitory potency which can be administered at low doses.

## Additional information

Additional methods and figure legends are provided in Additional file 7.

## Additional files

Additional file 1: Figure S6. Stable downregulation of TBK1 is associated with decreased LPS-induced c-fos activity. (A) Western blot showing TBK expression in RAW264.7 macrophages stably transduced with scrambled or TBK1-specific shRNA during the time course of LPS-incubation. The blots show a representative result, the diagram shows the densitometric analysis of four independent experiments. (B) c-fos transcription factor activity in nuclear extracts of RAW264.7 cells stably transduced with scrambled shRNA or TBK1 shRNA, respectively, as assessed by TransAM transcription factor ELISA (*n* = 3); black columns = scrambled shRNA; light grey columns = TBK1 shRNA, Univariate ANOVA with Bonferroni post-hoc analysis **P* < 0.05 and ****P* < 0.001 in comparison to untreated control, ^#^ *P* < 0.05, significant mean difference between scrambled shRNA and TBK1 shRNA. Additional file 2: Figure S1. Antibody specificity. Western Blot analysis (A) and immunofluorescence ((B): dorsal spinal cord, (C): DRG) of TBK1 in different mouse genotypes to confirm specificity of the antibody. Scale Bar: 10 μm. Additional file 3: Figure S2. Regulation of TBK1 in primary immune cells after inflammatory stimulation. Regulation of TBK1 mRNA in primary astrocytes (A) and microglia (B) after stimulation with a cytokine mix (TNF-α, 5 ng/ml; IL-1β, 1 ng/ml; LPS, 1 μg/ml) for 6 and 24 h, respectively, analyzed by quantitative RT-PCR. *n* = 3 independent incubations/cell type. Univariate ANOVA with Bonferroni post-hoc analysis * *P* < 0.05, and **** P* < 0.001. Additional file 4: Figure S3. TBK1expression and regulation in the dorsal root ganglia. (A) Representative co-immunofluorescence showing TBK1 expression in the dorsal root ganglia (one of 3 independent experiment, *n* = 3 mice/group) in combination with cell markers of non-myelinated nociceptive afferents (IB4) (A) and large myelinated non-nociceptive neurons (NF200) (B), respectively. TBK1 was stained with Cy-3 (red), cell markers with Alexa Fluor 488 (green). The images show (from left to right side): TBK1 alone, cell marker alone, and merged (representative result from 3 independent experiments). Scale Bar: 20 μm. (C, D) Time course of the TBK1 mRNA expression in the dorsal root ganglia after peripheral injection of zymosan A (C) and formalin (D), respectively, (*n* = 3 mice/group). Additional file 5: Figure S4. Regulation of TBK1 and inflammatory cytokines in the paw. (A) TBK1 mRNA expression in the contra- and ipsilateral paws of wild type mice 48 h after zymosan injection. Student’s *t* test ****P < 0.001* significant mean difference between contra- and ipsilateral paws (*n* = 3 mice/group). (B-D) Regulation of inflammatory cytokines (B: IL-1β, C: IL-6, D: IFN-β1) in the contra- and ipsilateral paws of wild type (black columns), TNFR^−/−^(dark grey columns) and TBK1^−/−^/TNFR^−/−^mice (light grey columns) 48 h after zymosan injection. Univariate ANOVA with Bonferroni post-hoc analysis, ****P < 0.001* significant mean difference compared to contralateral paw (*n* = 3 mice/group). Additional file 6: Figure S5. Effects of celecoxib on zymosan-induced hyperalgesia in mice with different genotypes. Time course of mechanical hyperalgesia in wild type (A), TNFR^−/−^mice (B) and TBK1^−/−^/TNFR^−/−^mice (C) with and without administration of celecoxib (10 mg/kg BW, p.o.) 30 min prior to zymosan A injection. The diagram shows the delta paw withdrawal latencies (ΔPWL) in response to mechanical stimulation as assessed with a Dynamic Plantar Aesthesiometer (*n* = 6 mice/group (D)). Comparison of the area under the paw withdrawal latency versus time curve between wild type (black column), TNFR^−/−^(dark grey column) and TBK1^−/−^/TNFR^−/−^(light grey column) mice 3 to 7 h after zymosan A injection. Univariate ANOVA with Bonferroni post-hoc analysis, **P < 0.05* significant mean difference between celecoxib/zymosan treated groups and zymosan treated controls. ^#^ *P* < 0.05 significant mean difference between the genotypes. Additional file 7:
**Additional methods and figure legends.**
