# Supplementary material for: TANK-binding kinase 1 (TBK1) modulates inflammatory hyperalgesia by regulating MAP kinases and NF-κB dependent genes
Source: J Neuroinflammation. 2015 May 23;12:100. doi: 10.1186/s12974-015-0319-3 (PMC4449530; doi:10.1186/s12974-015-0319-3)

Suppl. Figure 6: Stable downregulation of TBK1 is associated with decreased LPS-induced c-fos activity

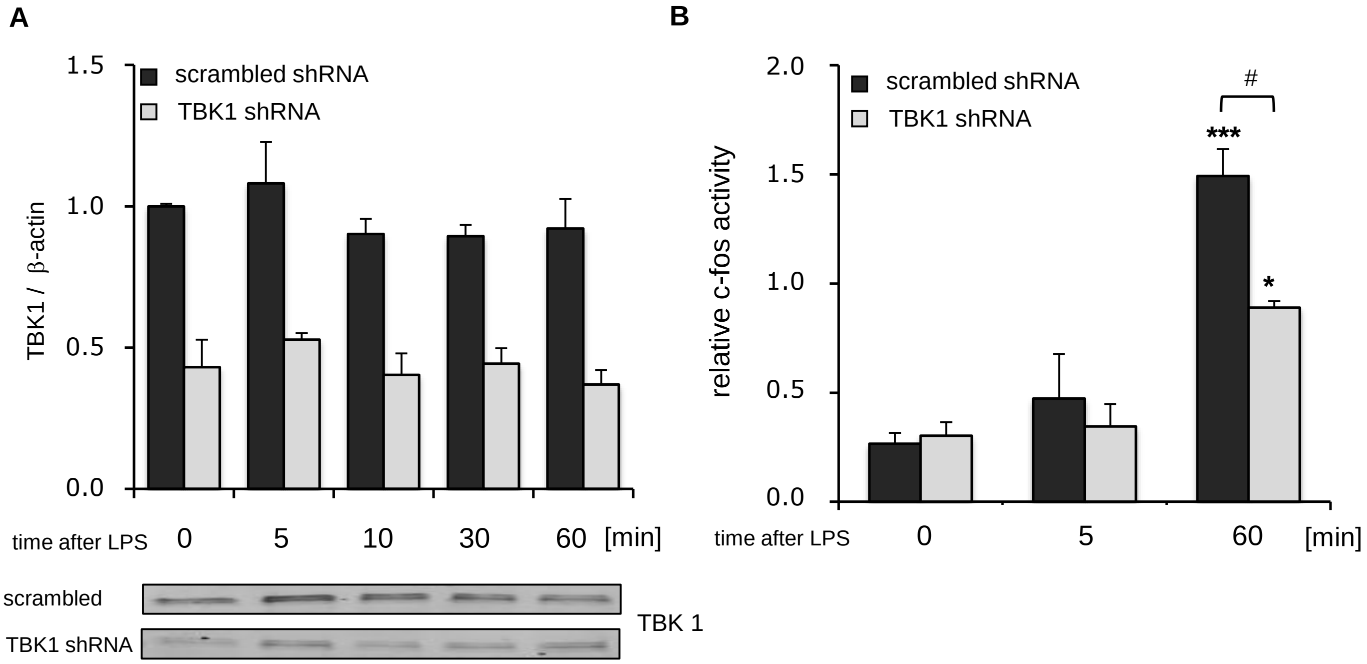

Supplement: Additional file 1: Figure S6. — Stable downregulation of TBK1 is associated with decreased LPS-induced c-fos activity. (A) Western blot showing TBK expression in RAW264.7 macrophages stably transduced with scrambled or TBK1-specific shRNA during the time course of LPS-incubation. The blots show a representative result, the diagram shows the densitometric analysis of four independent experiments. (B) c-fos transcription factor activity in nuclear extracts of RAW264.7 cells stably transduced with scrambled shRNA or TBK1 shRNA, respectively, as assessed by TransAM transcription factor ELISA (n = 3); black columns = scrambled shRNA; light grey columns = TBK1 shRNA, Univariate ANOVA with Bonferroni post-hoc analysis *P < 0.05 and ***P < 0.001 in comparison to untreated control, # P < 0.05, significant mean difference between scrambled shRNA and TBK1 shRNA. [file 12974_2015_319_MOESM1_ESM.pdf]
