# Supplementary material for: TANK-binding kinase 1 (TBK1) modulates inflammatory hyperalgesia by regulating MAP kinases and NF-κB dependent genes
Source: J Neuroinflammation. 2015 May 23;12:100. doi: 10.1186/s12974-015-0319-3 (PMC4449530; doi:10.1186/s12974-015-0319-3)

Suppl. Figure 1: Western Blot analysis (A) and immunofluorescence (B, C) of TBK1 in different mouse genotypes to confirm specificity of the antibody

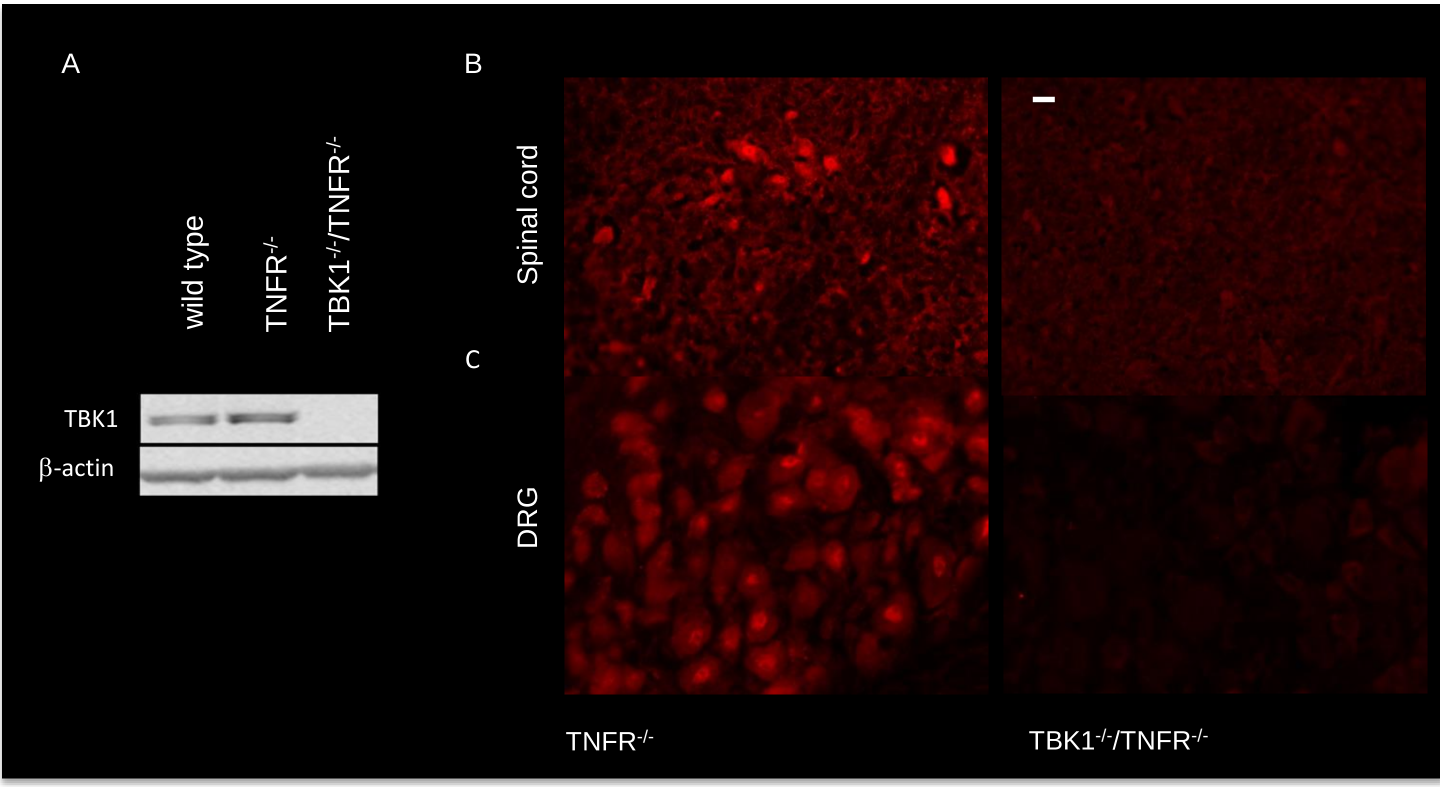

Supplement: Additional file 2: Figure S1. — Antibody specificity. Western Blot analysis (A) and immunofluorescence ((B): dorsal spinal cord, (C): DRG) of TBK1 in different mouse genotypes to confirm specificity of the antibody. Scale Bar: 10 μm. [file 12974_2015_319_MOESM2_ESM.pdf]
