# Supplementary figures and images for: TANK-binding kinase 1 (TBK1) modulates inflammatory hyperalgesia by regulating MAP kinases and NF-κB dependent genes
Source: J Neuroinflammation. 2015 May 23;12:100. doi: 10.1186/s12974-015-0319-3 (PMC4449530; doi:10.1186/s12974-015-0319-3)

**Suppl. Figure 2:** Regulation of TBK1 in primary immune cells after inflammatory stimulation

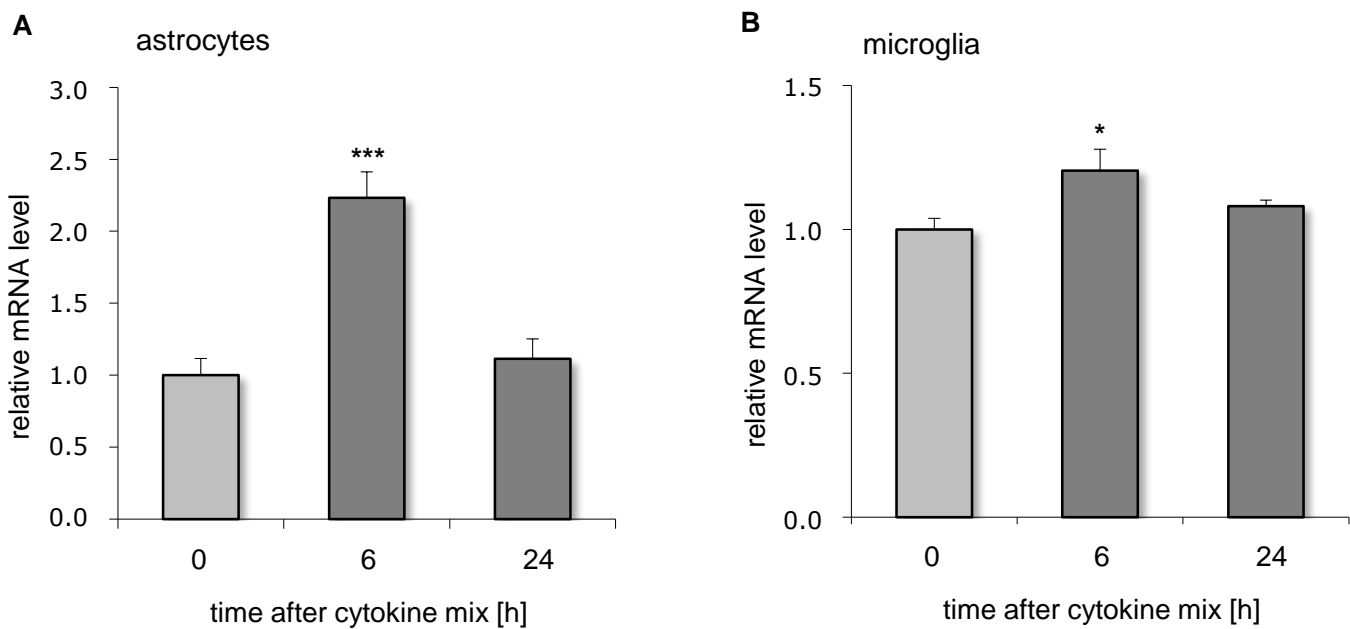

Supplement: Additional file 3: Figure S2. — Regulation of TBK1 in primary immune cells after inflammatory stimulation. Regulation of TBK1 mRNA in primary astrocytes (A) and microglia (B) after stimulation with a cytokine mix (TNF-α, 5 ng/ml; IL-1β, 1 ng/ml; LPS, 1 μg/ml) for 6 and 24 h, respectively, analyzed by quantitative RT-PCR. n = 3 independent incubations/cell type. Univariate ANOVA with Bonferroni post-hoc analysis * P < 0.05, and *** P < 0.001. [file 12974_2015_319_MOESM3_ESM.pdf]

**Suppl. Figure 3: TBK1 expression and regulation in the DRGs**

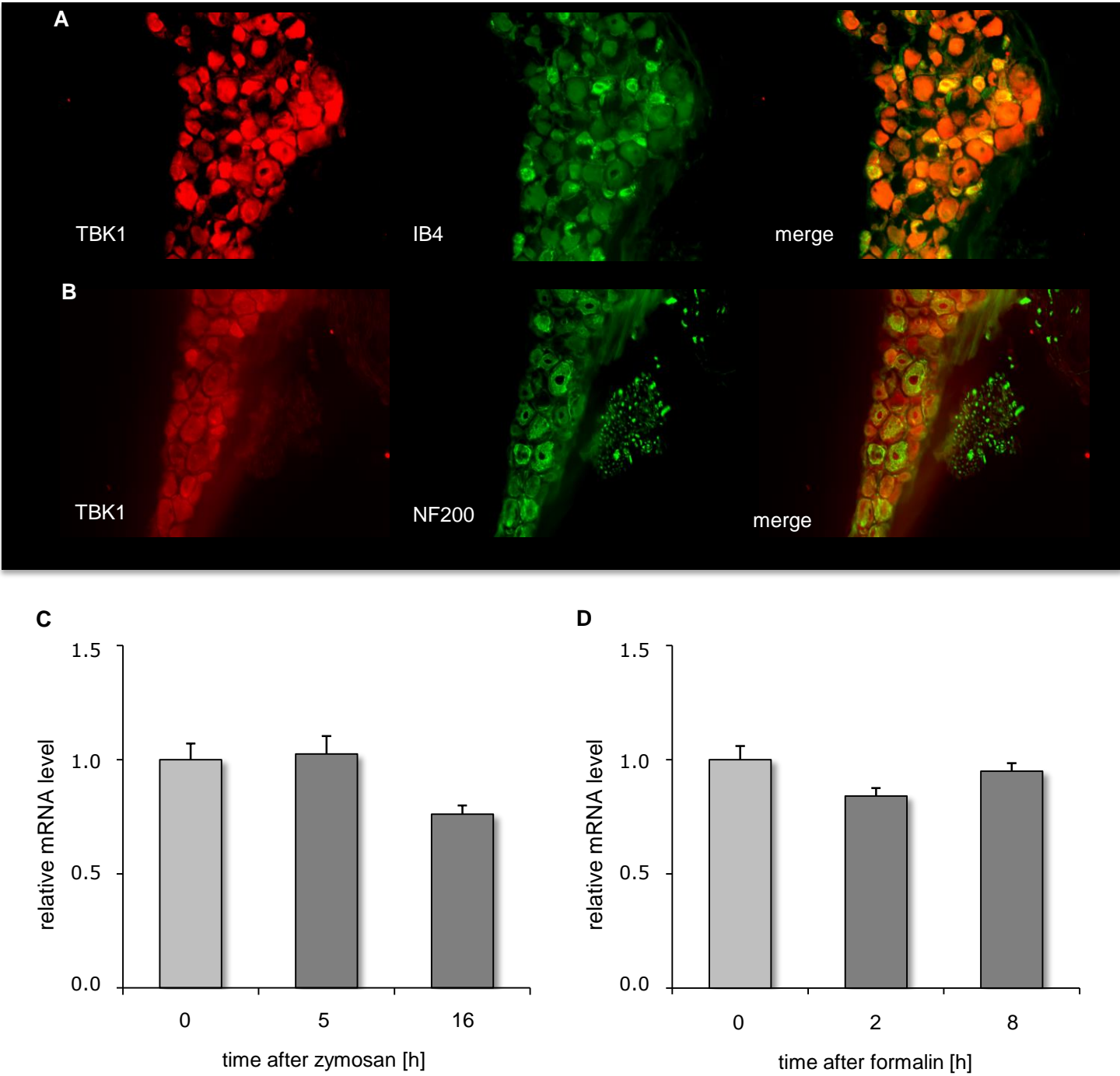

Supplement: Additional file 4: Figure S3. — TBK1expression and regulation in the dorsal root ganglia. (A) Representative co-immunofluorescence showing TBK1 expression in the dorsal root ganglia (one of 3 independent experiment, n = 3 mice/group) in combination with cell markers of non-myelinated nociceptive afferents (IB4) (A) and large myelinated non-nociceptive neurons (NF200) (B), respectively. TBK1 was stained with Cy-3 (red), cell markers with Alexa Fluor 488 (green). The images show (from left to right side): TBK1 alone, cell marker alone, and merged (representative result from 3 independent experiments). Scale Bar: 20 μm. (C, D) Time course of the TBK1 mRNA expression in the dorsal root ganglia after peripheral injection of zymosan A (C) and formalin (D), respectively, (n = 3 mice/group). [file 12974_2015_319_MOESM4_ESM.pdf]

**Suppl. Figure 4:** Regulation of TBK1 and inflammatory cytokines in the paw

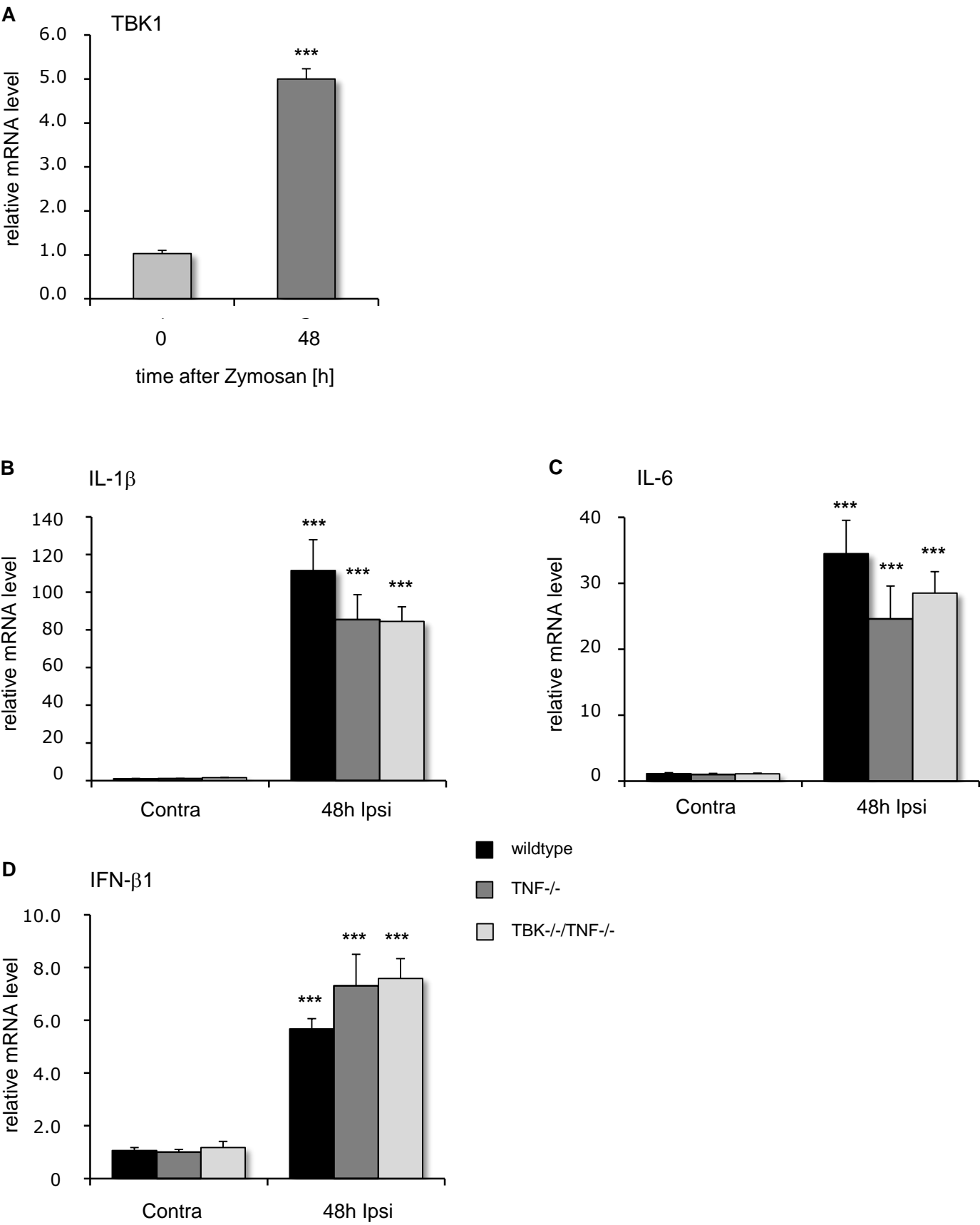

Supplement: Additional file 5: Figure S4. — Regulation of TBK1 and inflammatory cytokines in the paw. (A) TBK1 mRNA expression in the contra- and ipsilateral paws of wild type mice 48 h after zymosan injection. Student’s t test ***P < 0.001 significant mean difference between contra- and ipsilateral paws (n = 3 mice/group). (B-D) Regulation of inflammatory cytokines (B: IL-1β, C: IL-6, D: IFN-β1) in the contra- and ipsilateral paws of wild type (black columns), TNFR−/−(dark grey columns) and TBK1−/−/TNFR−/−mice (light grey columns) 48 h after zymosan injection. Univariate ANOVA with Bonferroni post-hoc analysis, ***P < 0.001 significant mean difference compared to contralateral paw (n = 3 mice/group). [file 12974_2015_319_MOESM5_ESM.pdf]
