# Supplementary material for: TANK-binding kinase 1 (TBK1) modulates inflammatory hyperalgesia by regulating MAP kinases and NF-κB dependent genes
Source: J Neuroinflammation. 2015 May 23;12:100. doi: 10.1186/s12974-015-0319-3 (PMC4449530; doi:10.1186/s12974-015-0319-3)

Suppl. Figure 5: Effects of celecoxib on zymosan-induced hyperalgesia in mice with different genotypes

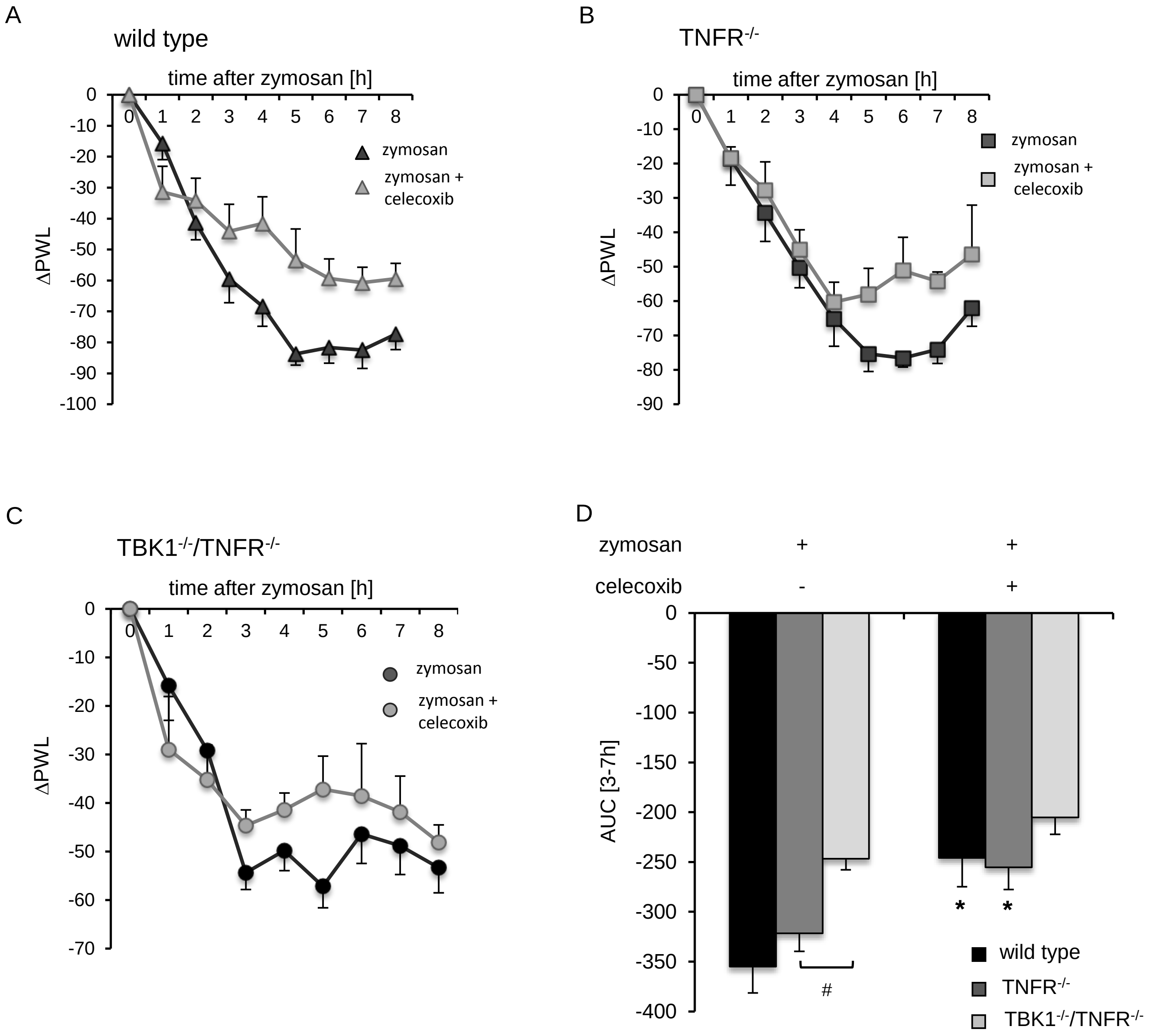

Supplement: Additional file 6: Figure S5. — Effects of celecoxib on zymosan-induced hyperalgesia in mice with different genotypes. Time course of mechanical hyperalgesia in wild type (A), TNFR−/−mice (B) and TBK1−/−/TNFR−/−mice (C) with and without administration of celecoxib (10 mg/kg BW, p.o.) 30 min prior to zymosan A injection. The diagram shows the delta paw withdrawal latencies (ΔPWL) in response to mechanical stimulation as assessed with a Dynamic Plantar Aesthesiometer (n = 6 mice/group (D)). Comparison of the area under the paw withdrawal latency versus time curve between wild type (black column), TNFR−/−(dark grey column) and TBK1−/−/TNFR−/−(light grey column) mice 3 to 7 h after zymosan A injection. Univariate ANOVA with Bonferroni post-hoc analysis, *P < 0.05 significant mean difference between celecoxib/zymosan treated groups and zymosan treated controls. # P < 0.05 significant mean difference between the genotypes. [file 12974_2015_319_MOESM6_ESM.pdf]
