# Supplementary material for: TANK-binding kinase 1 (TBK1) modulates inflammatory hyperalgesia by regulating MAP kinases and NF-κB dependent genes
Source: J Neuroinflammation. 2015 May 23;12:100. doi: 10.1186/s12974-015-0319-3 (PMC4449530; doi:10.1186/s12974-015-0319-3)

**Supplement**

**Methods**

## Preparation of primary cells

### Astrocytes.

Primary astrocytes were prepared from embryonic mouse brain (E15). Brains were dissected out, washed in HBSS, and digested for 2 h in 10 % trypsin solution at 37 °C. After two washes with DMEM containing 10 % FCS / 1 % penicillin/streptomycin, the brains were homogenized and plated onto culture dishes in DMEM with the addition of 10 % FCS/ 1 % penicillin/streptomycin and 1 % 200 mM L-glutamine. The culture was then incubated for 10-14 days until microglia could be shaken off and separated from the astrocytes. The remaining astrocyte cultures were used for two or three passages and submitted to cytokine-stimulation.

### Microglia.

For primary microglia cultures, embryonic mouse brains (E15) were dissected, washed in HBSS, and digested for 2 h in 10 % trypsin solution at 37 °C. After two washes with DMEM containing 10 % FCS/ 1 % penicillin/streptomycin, the brains were homogenized and plated onto culture dishes in DMEM with the addition of 10 % FCS/ 1 % penicillin/streptomycin and 1 % 200 mM L-glutamine. The culture was then incubated for 10-14 days until microglia could be shaken off. Afterwards, cells were plated onto new dishes and submitted to cytokine-stimulation experiments.

## Real-time PCR (Taqman)

RNA was prepared from the ipsi- and contralateral paw of zymosan-injected mice after 48 h. Quantitative RT-PCR was performed as described in material and methods. Expression of IL-1β, IL-6 and IFN-β1 was assessed related to GAPDH mRNA. The following gene-specific primers were used:

IL-1β FW 5’-CTGGTGTGTGACGTTCCCATTA-3’

RV 5’-CCGACAGCACGAGGCTTT-3’

IL-6: FW 5’- GAGGATACCACTCCCAACAGACC -3’

RV 5’- AAGTGCATCATCGTTGTTCATACA -3’

IFN-β1: FW 5’- TTACACTGCCTTTGCCATCC -3’

RV 5’- ACTGTCTGCTGGTGGAGTTCAT -3’

*Effects of COX-2 inhibition by celecoxib in the zymosan induced paw inflammation*

Celecoxib (10 mg/kg body weight, p.o.) was administered to wild type (C57BL/6), TNFR^-/-^ and TBK1^-/-^/TNFR^-/-^ mice 30 min prior to injection of zymosan. Then, zymosan induced mechanical hyperalgesia was analyzed, as described in the methods section.

**Supplemental Figures**

***Supplemental Figure 1:*** *Antibody specificity*

Western Blot analysis (A) and immunofluorescence (B: dorsal spinal cord, C: DRG) of TBK1 in different mouse genotypes to confirm specificity of the antibody. Scale Bar: 10 µm.

***Supplemental Figure 2:*** *Regulation of TBK1 in primary immune cells after inflammatory stimulation*

Regulation of TBK1 mRNA in primary astrocytes (A) and microglia (B) after stimulation with a cytokine mix (TNF-α, 5 ng/ml; IL-1β, 1 ng/ml; LPS, 1 µg/ml) for 6 and 24 h, respectively, analyzed by quantitative RT-PCR. n=3 independent incubations/cell type. Univariate ANOVA with Bonferroni post-hoc analysis * *P*<0.05, and **** P*<0.001.

***Supplemental Figure 3:*** *TBK1expression and regulation in the dorsal root ganglia*

(A) Representative co-immunofluorescence showing TBK1 expression in the dorsal root ganglia (one of 3 independent experiment, n=3 mice/group) in combination with cell markers of non-myelinated nociceptive afferents (IB4) (A) and large myelinated non-nociceptive neurons (NF200) (B), respectively. TBK1 was stained with Cy-3 (red), cell markers with Alexa Fluor 488 (green). The images show (from left to right side): TBK1 alone, cell marker alone and merged (representative result from 3 independent experiments). Scale Bar: 20 µm. (C, D) Time course of the TBK1 mRNA expression in the dorsal root ganglia after peripheral injection of zymosan A (C) and formalin (D), respectively, (n=3 mice/group).

***Supplemental Figure 4:*** *Regulation of TBK1 and inflammatory cytokines in the paw*

(A) TBK1 mRNA expression in the contra- and ipsilateral paws of wild type mice 48 h after zymosan injection. Students t-test ****P< 0.001* significant mean difference between contra- and ipsilateral paws (n=3 mice/group). (B-D) Regulation of inflammatory cytokines (B: IL-1β, C: IL-6, D: IFN-β1) in the contra- and ipsilateral paws of wild type (black columns), TNFR^-/-^ (dark grey columns) and TBK1^-/-^/TNFR^-/-^ mice (light grey columns) 48 h after zymosan injection. Univariate ANOVA with Bonferroni post-hoc analysis, ****P<0.001* significant mean difference compared to contralateral paw (n=3 mice/group).

***Supplemental Figure 5:*** *Effects of celecoxib on zymosan-induced hyperalgesia in mice with different genotypes*

(A) Time course of mechanical hyperalgesia in wild type (A), TNFR^-/-^ mice (B) and TBK1^-/-^/TNFR^-/-^ mice (C) with and without administration of celecoxib (10 mg/kg BW, p.o.) 30 min prior to zymosan A injection. The diagram shows the delta paw withdrawal latencies (ΔPWL) in response to mechanical stimulation as assessed with a Dynamic Plantar Aesthesiometer (n=6 mice/group (D)). Comparison of the area under the paw withdrawal latency versus time curve between wild type (black column), TNFR^-/-^ (dark grey column) and TBK1^-/-^/TNFR^-/-^ (light grey column) mice 3 to 7 hours after zymosan A injection. Univariate ANOVA with Bonferroni post-hoc analysis, **P<0.05* significant mean difference between celecoxib/zymosan treated groups and zymosan treated controls. ^#^*P*<0.05 significant mean difference between the genotypes.

***Supplemental Figure 6:*** *Stable downregulation of TBK1 is associated with decreased LPS-induced c-fos activity*

(A) Western blot showing TBK expression in RAW264.7 macrophages stably transduced with scrambled or TBK1-specific shRNA during the time course of LPS-incubation. The blots show a representative result, the diagram shows the densitometric analysis of four independent experiments. (B) c-fos transcription factor activity in nuclear extracts of RAW264.7 cells stably transduced with scrambled shRNA or TBK1 shRNA, respectively, as assessed by TransAM transcription factor ELISA (n=3); black columns = scrambled shRNA; light grey columns = TBK1 shRNA, Univariate ANOVA with Bonferroni post-hoc analysis **P*<0.05 and ****P*<0.001 in comparison to untreated control, ^#^*P*<0.05, significant mean difference between scrambled shRNA and TBK1 shRNA.


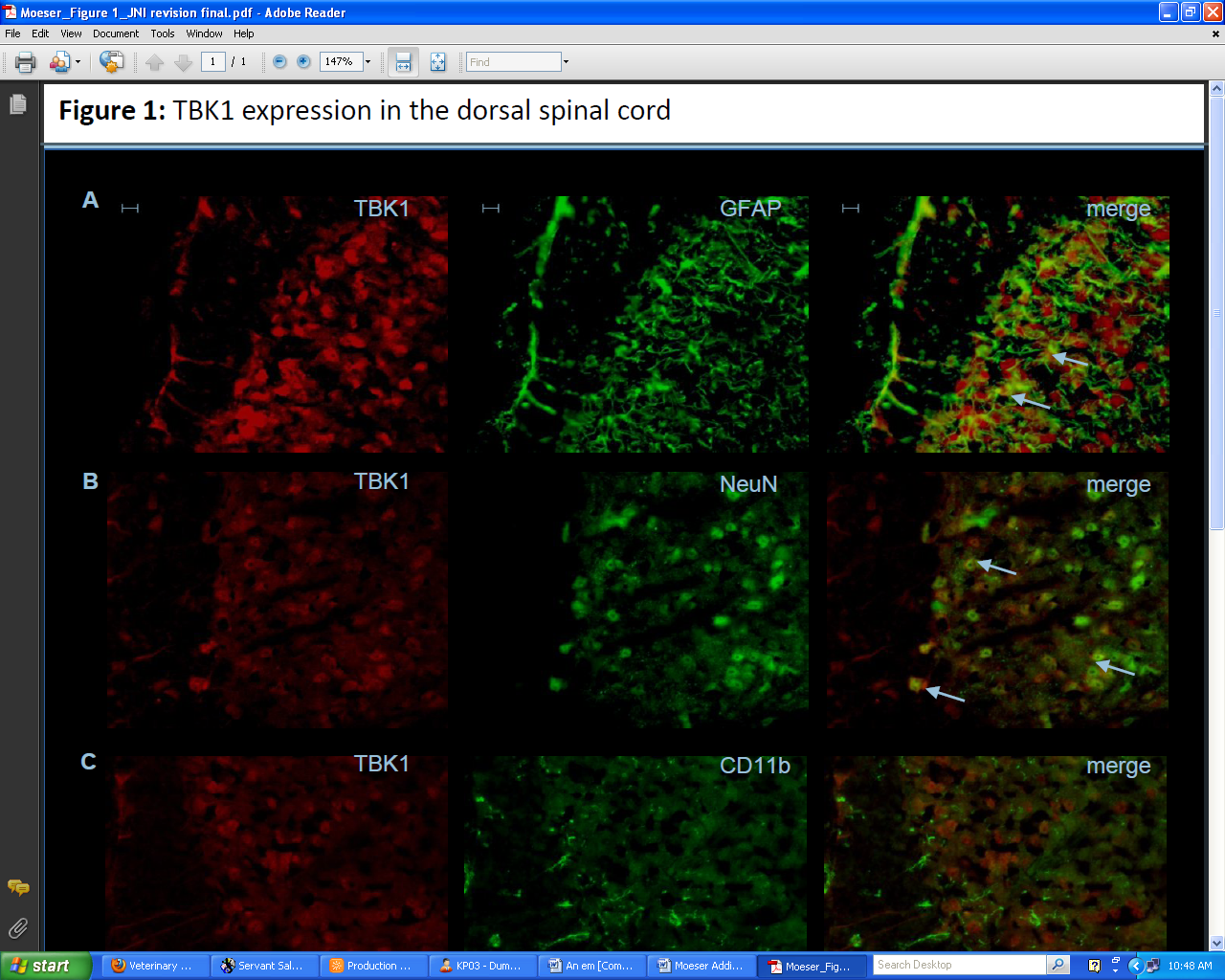


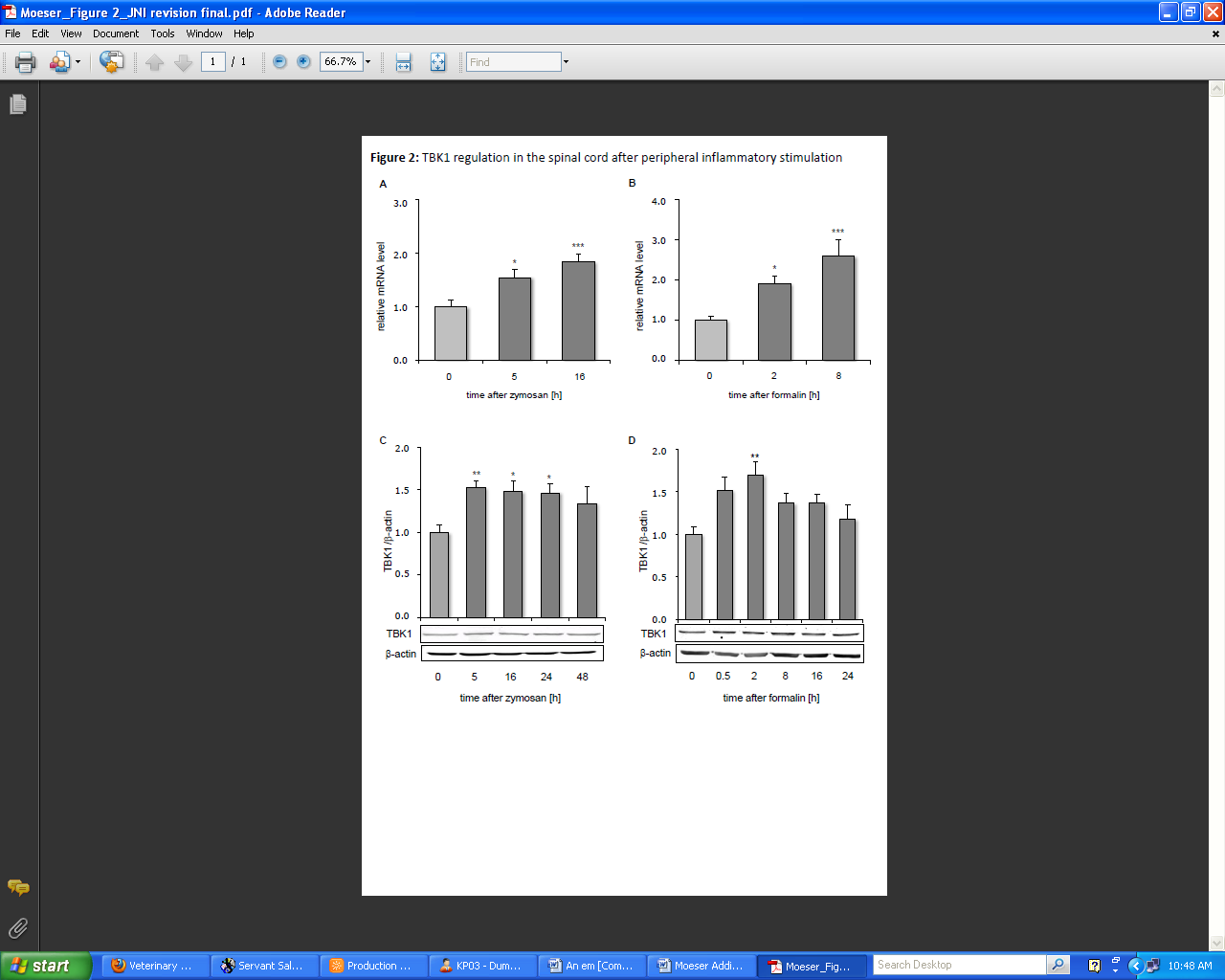


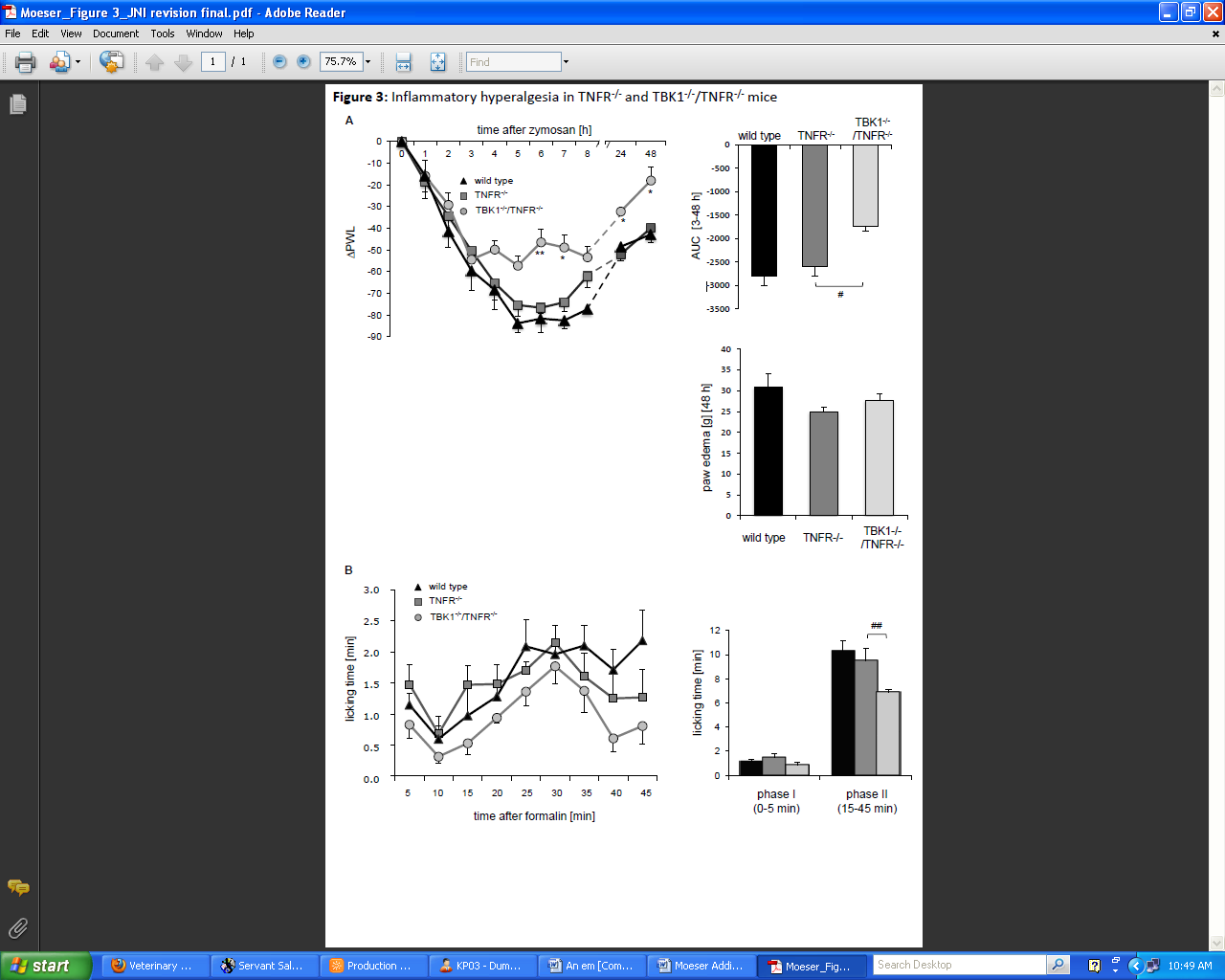


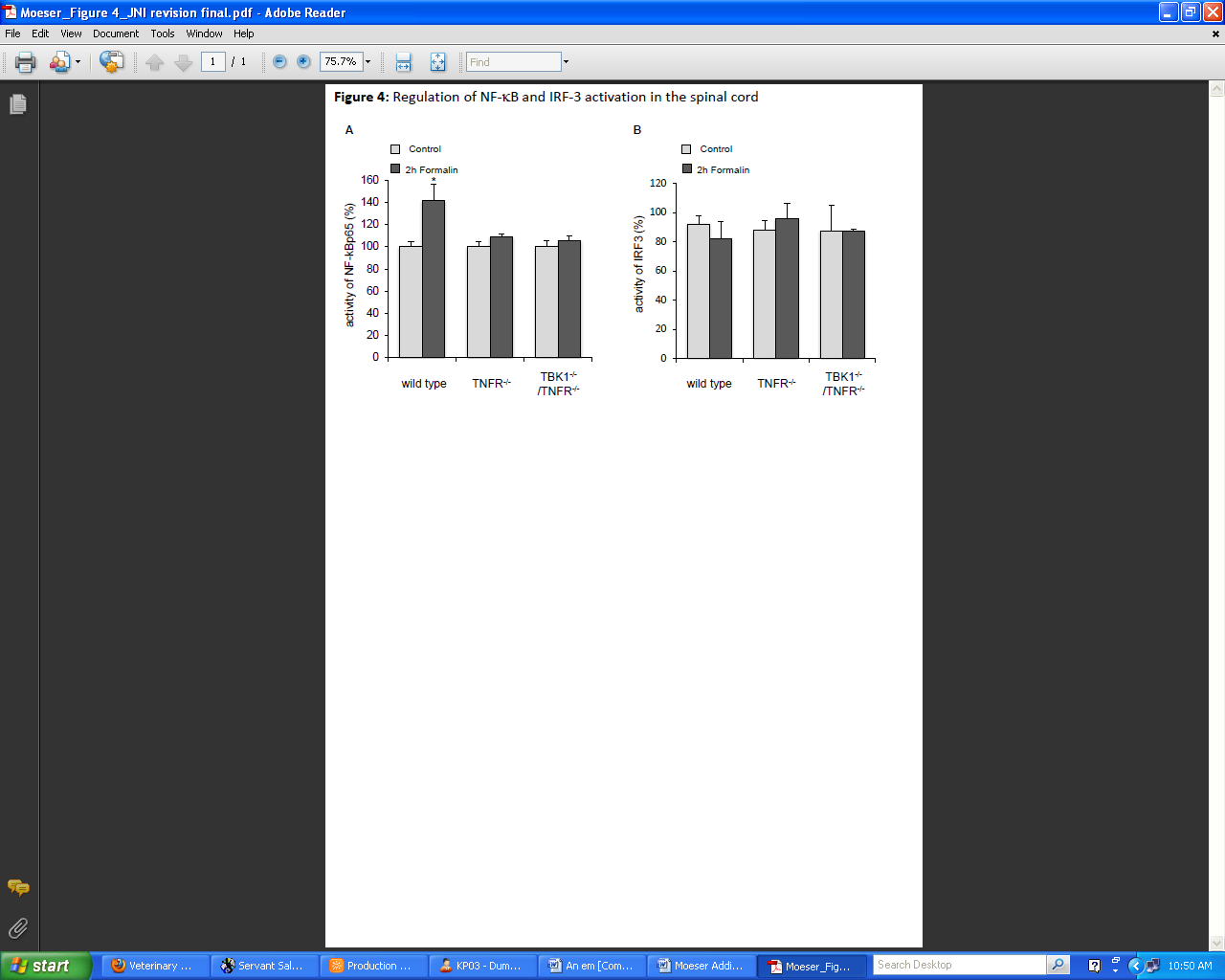


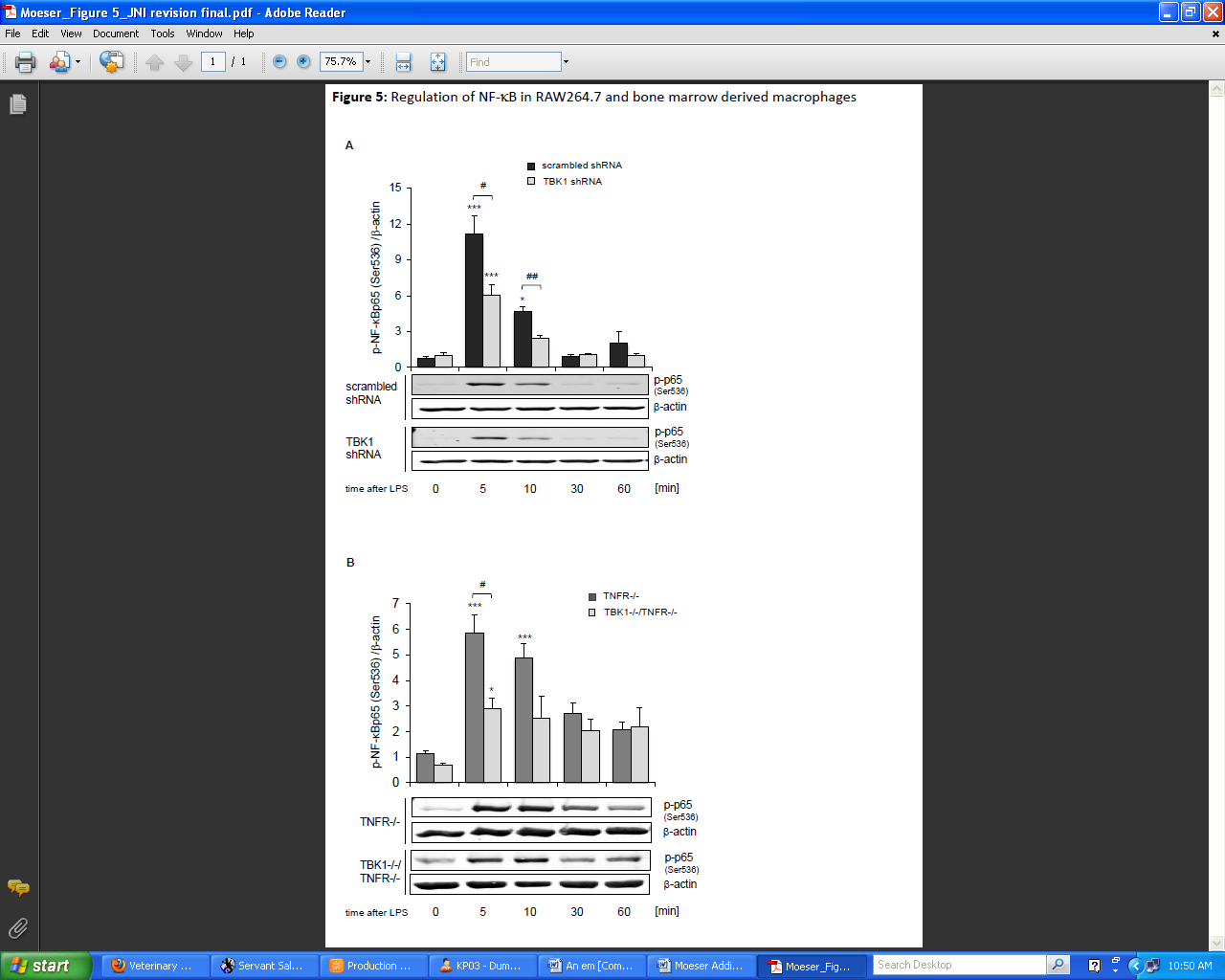


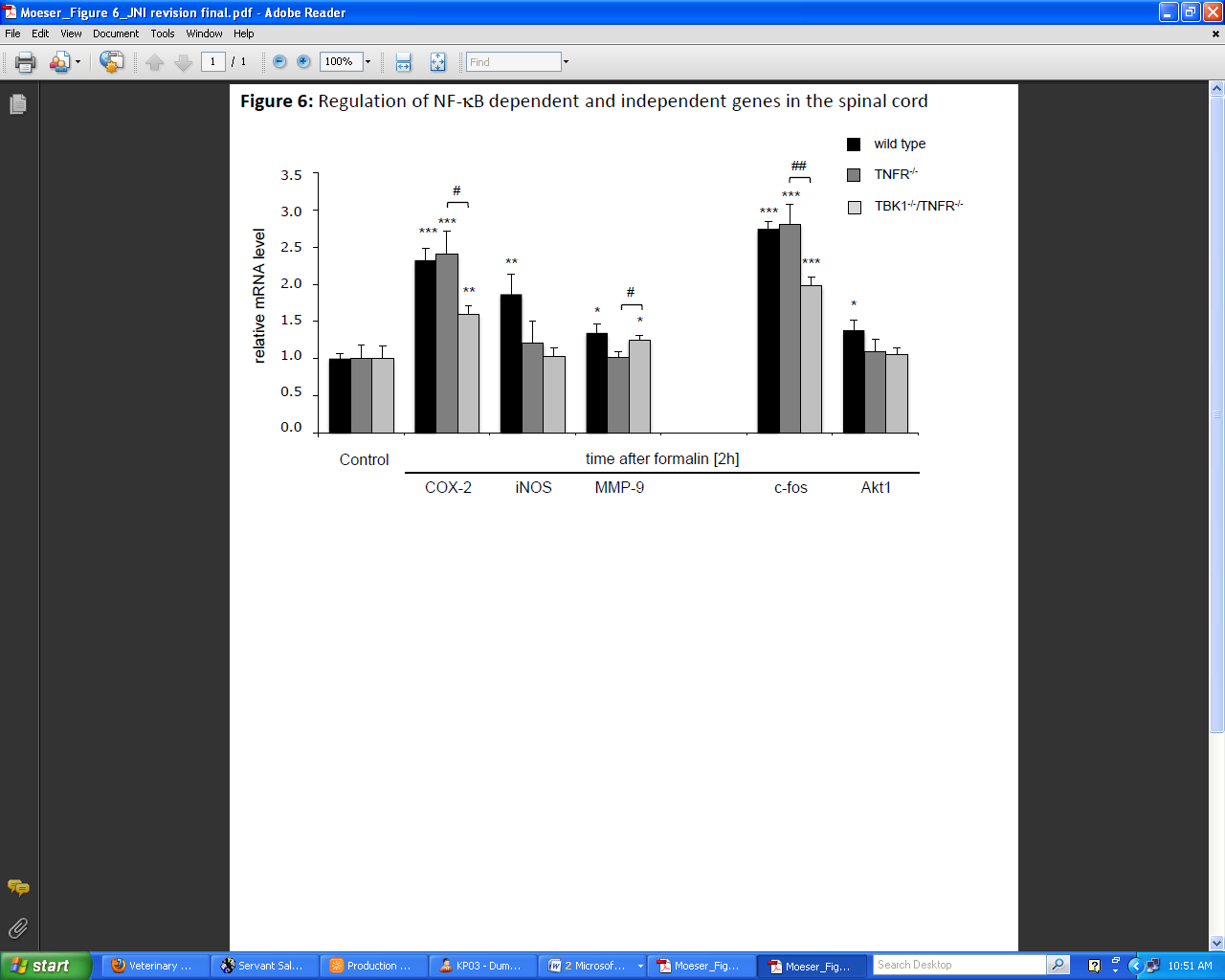


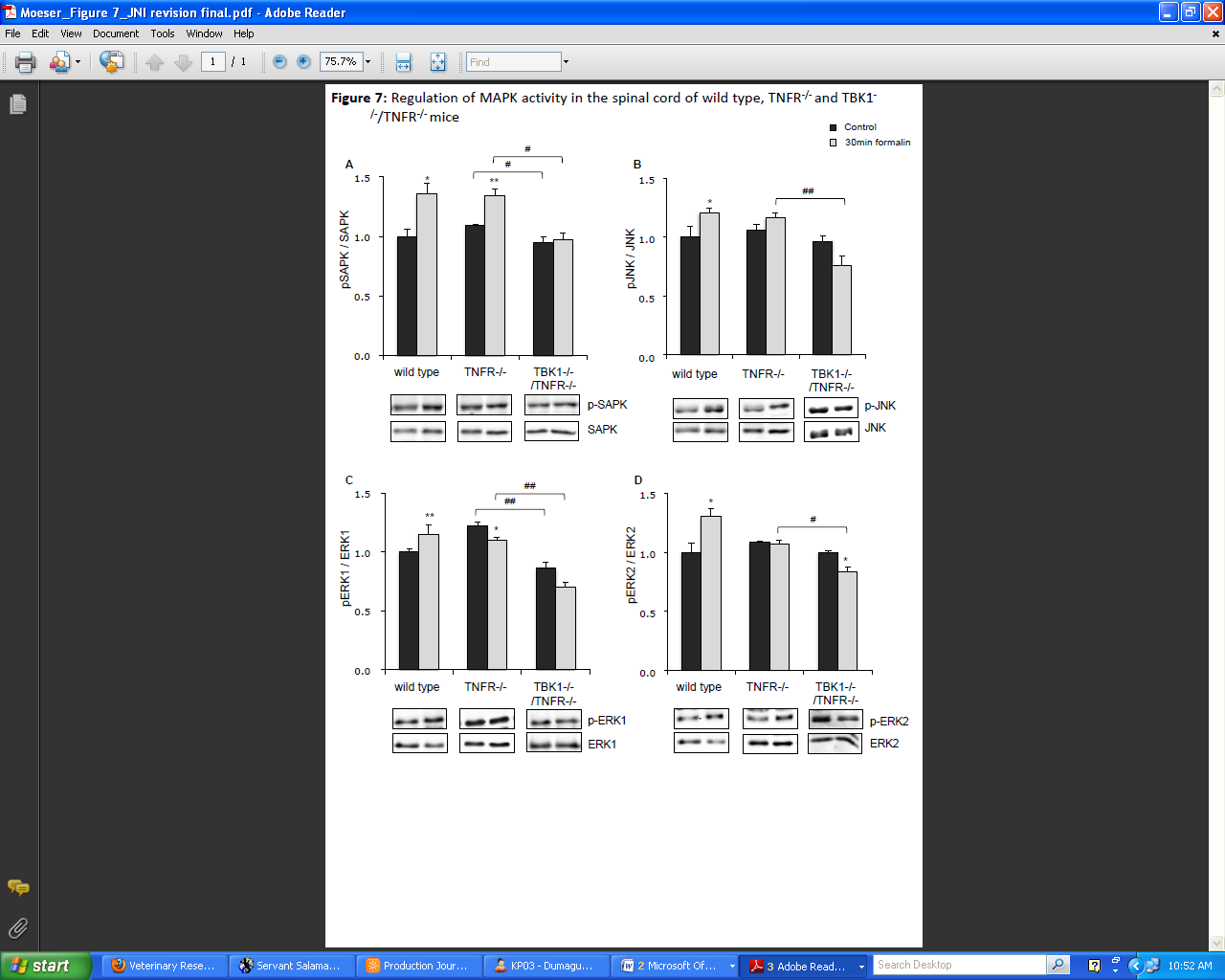

Supplement: Additional file 7: — Additional methods and figure legends. [file 12974_2015_319_MOESM7_ESM.docx]
